# Systematic review of the global epidemiology, clinical and laboratory profile of enteric fever

**DOI:** 10.7189/jogh.05.020407

**Published:** 2015-12

**Authors:** Asma Azmatullah, Farah Naz Qamar, Durrane Thaver, Anita KM Zaidi, Zulfiqar A Bhutta

**Affiliations:** 1 Department of Paediatrics and Child Health, Aga Khan University, Karachi, Pakistan; 2 Centre for Global Child Health, Research Institute, The Hospital for Sick Children, Toronto, Canada; 3 Centre of Excellence in Women & Child Health, The Aga Khan University, Karachi, Pakistan

## Abstract

**Background:**

Children suffer the highest burden of enteric fever among populations in South Asian countries. The clinical features are non–specific, vary in populations, and are often difficult to distinguish clinically from other febrile illnesses, leading to delayed or inappropriate diagnosis and treatment. We undertook a systematic review to assess the clinical profile and laboratory features of enteric fever across age groups, economic regions, level of care and antibiotic susceptibility patterns.

**Methods:**

We searched PubMed (January 1964–December 2013) for studies describing clinical features in defined cohorts of patients over varying time periods. Studies with all culture–confirmed cases or those with at least 50% culture–confirmed cases were included. 242 reports were screened out of 4398 relevant articles and 180 reports were included for final review.

**Results:**

96% of studies were from an urban location, 96% were hospital–based studies, with 41% of studies were from South Asia. Common clinical features in hospitalized children include high–grade fever, coated tongue, anaemia, nausea/vomiting, diarrhea, constipation, hepatomegaly, splenomegaly neutrophilia, abdominal distension and GI bleeding. In adults’ nausea/vomiting, thrombocytopenia and GI perforation predominate. The case–fatality rate in children under 5 years is higher than school aged children and adolescents, and is highest in Sub Saharan Africa and North Africa/Middle East regions. Multi–drug resistant enteric fever has higher rates of complications than drug sensitive enteric fever, but case fatality rates were comparable in both.

**Conclusions:**

Our findings indicate variability in disease presentation in adults compared to children, in different regions and in resistant vs sensitive cases. Majority of studies are from hospitalized cases, and are not disaggregated by age. Despite higher complications in MDR enteric fever, case fatality rate is comparable to sensitive cases, with an overall hospital based CFR of 2%, which is similar to recent global estimates. This review underscores the importance of further epidemiological studies in community settings among children and adults, and the need for further preventable measures to curtail the burden of disease.

Enteric fever, representing a systemic infection caused by *Salmonella enteric* serovar Typhi (*S*. *typhi*) and *Salmonella enterica* serovar Paratyphi (*S. paratyphi*), is a common cause of morbidity in the developing world, particularly in South and South East Asia [1,2]. It is estimated that over 22 million cases and more than 200 000 deaths of typhoid fever occurred in the year 2000, with the majority of disease burden being borne by children and adolescents in South and South–East Asia [1]. Highest incidence has been documented in impoverished, overcrowded areas with poor access to sanitation such as the urban slum areas of North Jakarta (Indonesia), Kolkata (India) and Karachi (Pakistan) with annual incidence rates of blood culture–confirmed enteric fever ranging from 180–494/100 000 among 5–15 year-olds and 140–573/100 000 among those 2–4 years old [3]. However, it is recognized that the assessment of disease burden from Africa remains uncertain, with recent reports suggesting that it may be an increasingly recognized but underreported problem, requiring further prevalence studies [4-6]. Prevalences ranging from 0% to 4.23% have been reported from Kenya, Africa, in a recent review [7].

Despite the high burden of disease, challenges in the diagnosis and management of enteric remain. Clinical diagnosis of enteric fever is nonspecific and mimics other febrile illnesses like malaria and dengue fever and influenza [5,6]. This is particularly true for children who can present with atypical signs and complications such as neurological dysfunction, nephropathy and cardiac abnormalities [4,8,9] and thus lead the clinician away from a diagnosis of enteric fever. Attempts have been made to develop and validate clinical algorithms [10,11], without becoming mainstream for usage in diagnosis. The lack of availability of the blood cultures, in many small hospitals and community settings in endemic populations is an additional limitation, as is the low yield of the test due to prior antibiotic treatment or sampling issues in young children [12,13]. These factors can contribute to delayed diagnosis and/or inappropriate treatment [12,14].

The emergence of drug resistance and changing patterns of both multi–drug (MDR)(resistant to all three traditional first–line agents: chloramphenicol; ampicillin; and co–trimoxazole) and fluoroquinolone resistant *S*. *typhi* and *S*. *paratyphi* [12,15] has been associated with reported changes in the severity and clinical profile of enteric fever [6,16-19].Nearly 60% of typhoid fever isolates tested in Kolkata and Karachi and 44% of those in Hue, Vietnam were resistant to nalidixic acid; making these cases less responsive to commonly used second line agents such as ciprofloxacin and other fluoroquinolones [3,14]. This has not only narrowed the therapeutic options in high disease burden countries but has also lead to increased treatment costs, severity of illness, higher rates of complications and higher case fatality rates [6,14,17,20,21].

Although enteric fever is essentially a paediatric disease in South Asia, there is dearth of retrospective and prospective studies done in children with culture proven enteric fever in the global literature [22]. Furthermore, most studies on enteric fever represent hospitalized subjects and the differences in the clinical features and severity of the disease may also differ substantially from those not requiring hospitalization. Hospitalization rates of up to 2–40% among culture–confirmed ambulatory enteric cases were found in five different study sites in Asia [23], but data from those not hospitalized could represent a different disease severity and pattern. Differences in health seeking behavior of hospitalized vs community based subjects as well as differences in access may also limit generalization of available literature on clinical patterns of enteric fever [6].

In addition, reports suggest a considerable influence of age; with some studies documenting increased morbidity and mortality in younger children [19,20,23-25] while others [26,27] report comparatively better outcomes in this age group. Reports also suggest differences in presentation and outcomes between children and adults [19,20,28].Data from individual studies suggest a difference in clinical spectrum of disease amongst geographical locations in high–income and low and middle–income countries. In a report from an Ethiopian children's hospital (1984–1995), intestinal perforation occurred in 27 patients (25%) out of which 10 (37%) died [29]. During a similar time (1982–1995) in Taiwan, only 2/71 cases of intestinal perforation were reported in children [30].Prevalence of co–morbidities such as HIV, differences in antimicrobial resistance patterns, over–the–counter antibiotic availability, substandard antibiotic preparations, lack of pipe–borne portable water supply, health system functionality and health seeking behaviors all weigh in to the differences seen in disease spectrum, complications and mortality across regions.

No comprehensive systematic review exists describing the differences in clinical features of enteric fever and the frequency of its complications by various age groups. Further, the differences in clinical presentation by economic and geographical regions and by drug resistance patterns have not been systematically investigated.

This systematic review assesses the clinical profile of enteric fever across different regions and age groups (children vs adults). We also compare the epidemiology of enteric in hospitalized and community settings and in children infected with multi–drug resistant vs sensitive strains of *S*. *typhi*. Finally we describe the relationship between multidrug resistance patterns and case–fatality rates over time.

## METHODS

**Figure 1:**
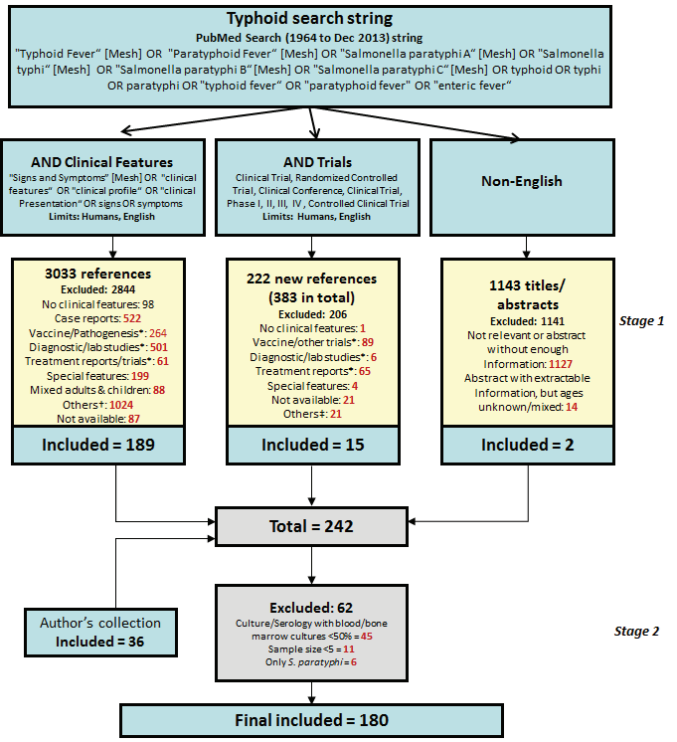
Search methodology. *Mixed ages, no clinical features or excluded complicated cases on enrollment. †“Others” (1024) includes: Studies on Typhoid carriers (44) Non-typhoid (mostly Rickettsia)/diarrheal diseases/other Salmonella (561) General public health/sanitation (58) Not relevant/other laboratory-based/miscellaneous (112) Reviews, letters, editorials (249). ‡“Others” (n = 20) includes: Non-typhoid(mostly Rickettsia)/diarrheal diseases/other Salmonella (n = 1), General public health/sanitation (n = 1), Not relevant/other laboratory-based/miscellaneous (n = 14), Reviews, letters, editorials (n = 5).

We searched PubMed for studies limited to Humans (1964 onwards; last searched December 2013), and English language using MeSH and text words as shown in **Figure 1**. We conducted additional parallel searches for the following to ensure comprehensive identification of all relevant reports: a) non–English language studies (title/abstracts screen); b) clinical trials; c) relevant articles were manually retrieved from reference lists and other pertinent studies, known to the authors and not already retrieved from PubMed were included (“author’s collection”).

All studies indicating documentation of clinical features, based on title and/or abstract, were retrieved in full–text where available (**Figure 1**). Regional break–up of countries was taken from the World Bank list of Economies (updated April 2012) [31].

### Inclusion criteria

We included studies which reported clinical features from diagnosed cases of enteric fever. Diagnosis was based on either a positive culture (blood, bone marrow, other sterile site–stool, urine) or a positive serological diagnostic test (Widal test/Typhi Dot test), as long as the diagnosis was confirmed by culture tests in at least 50% of these cases. Outcome data in children (age as author defined, or 0–15 years) or adults (age as author defined, or 12 years and above) was included if given in disaggregated form. We included clinical trials, vaccine trials, diagnostic studies, only where any clinical features were described provided they met the pre–defined criteria (**Box 1**).

Box 1 **Pre-set inclusion criteria**

1. Studies must have clearly documented data on at least one clinical feature (other than drug resistance or mortality)

2. Studies were included from 1964 onwards as well as articles not indexed in PubMed before that at the time of searching

3. Number of cases reported on in each study had to be more than 5 (n ≥5)

4. Studies with cases of only S. typhi were included, unless data was given for both *S. typhi* and *S. paratyphi* in an aggregated form which could not be separated

5. Studies must include enteric fever of all severity (eg: excluded if only uncomplicated cases were included)

6. Studies must have at least 50% or more culture positivity (blood, bone marrow, urine or stool) along with serologically positive cases (not included if only diagnosed on clinical basis)

7. Cases must be in distinct age groups–children or adults

8. Studies must not be from a certain population subset (eg, all HIV positive, all intestinal perforations)

9. Studies that were not in English must have adequate, extractable information in the abstract

10. Studies published from the same hospital/region and during the same time period were considered as duplicate/overlapping data and counted once using the largest reported denominator

### Exclusion criteria

We excluded case reports (as indexed, or those with a sample size ≤5), studies reporting mixed age groups (ie, 2 to 55 years) where disaggregation on age was not stated, with some or all cases diagnosed only on clinical suspicion and reports of selective patient groups (eg, all complicated, or all HIV cases, or all cases presenting with diarrhea). Studies using only a clinical diagnosis or serological diagnostic tests only (Widal test/Typhi Dot test), without culture confirmation were excluded. For studies reporting data for *S*. *typhi* and *S. paratyphi* separately, only data for *S*. *typhi* were extracted; however if studies did not present data separately, data was included as both *S*. *typhi* and *S. paratyphi*.

In addition to baseline characteristics, geographical location, resistance and clinical features, data were also extracted separately where available for different age groups and for multi–drug resistance and sensitive isolates. Clinical features were used as author defined or as a given set of definitions if otherwise undefined (Chart 1 in **Online Supplementary Documentation(Online Supplementary Document)**). For each clinical feature, we extracted the number of patients with the event and the number of patients assessed for the feature. Similar features were grouped together (such as “encephalopathy” and “lethargy” grouped under “altered mental status”); the largest uncombined numerator was used when several similar features were reported in a study.

### Statistical methods

Data was double entered into Microsoft Access 2007 and tabulated using Microsoft Excel 2007 (Microsoft Corp., Redmond, WA, USA) spreadsheets. Frequency tables of clinical features were calculated also using Microsoft Excel. Further analysis was done using χ^2^–testing for different ages (0–5 years vs 5–10 years; children 0–5 years and 5–10 years vs adults), for economical/geographical regions (Africa vs South Asia); for hospital vs community settings and for MDR strains vs sensitive strains). The level of significance was set at <0.05and odds ratio (OR) are reported for likelihood of clinical feature between different categories. All analysis was done using OpenEpi [32].

## RESULT

### Included studies

242 reports were screened out of a total of 4398 articles retrieved with the search strategy (Stage 1). All studies with culture (blood, bone marrow, other sterile site stool, urine) confirmed enteric fever were included, as well as serologically confirmed enteric fever if percentage of culture confirmed cases was more than 50% (Stage 2). Disaggregated age data from these studies, if available, were also extracted assuming a similar proportion of culture–confirmed cases in each age group. Categorization of excluded studies is shown in **Figure 1**.

**Figure 2:**
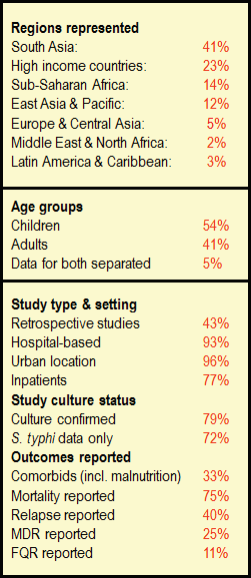
Breakdown of included studies. 8 studies with disaggregated data for Adults and Children are counted only once in Child category, 27 studies had overlapping/duplicate data (Total 180). MD – multidrug resistance; FQ – fluoroquinolones.

**Figure 3:**
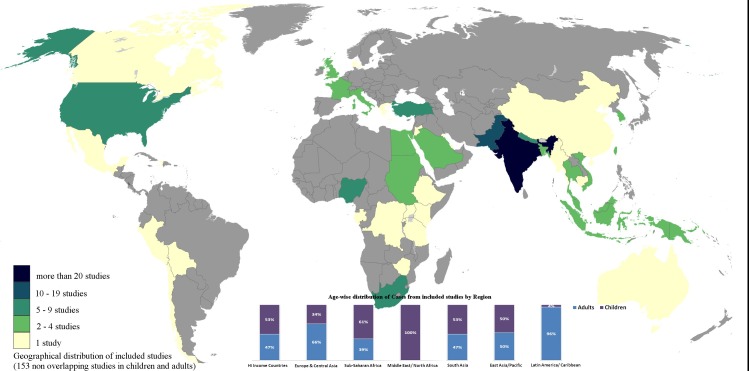
Map of geographical distribution of included studies.

A total of 180 reports were included for final review. **Figure 2** summarizes the characteristics of included studies (153 primary references and 27 references with overlapping data): 82 studies were on children, 63 on adults and 8 studies provided disaggregated data for adults and children (2 reports from overlapping or potentially overlapping data). Urban, hospital–based, inpatient retrospective studies were predominant. Data for resistance and relapse were uncommonly presented. Studies with only *S.typhi* were 72%, while 28% had representation of both *S. typhi* and *S*. *paratyphi* which could not be separated out. **Figure 3** shows the geographical representation of countries with included studies with the relative contribution of data from different regions. India far outranked other countries, with 46 studies in total (41% of included studies).

### Epidemiology of enteric fever in children vs adults

Studies in adults and those with data from children in disaggregated age groups were tabulated, from all available settings (**Table 1** and Table S1 in **Online Supplementary Document(Online Supplementary Document)**). Fever was universal (97%–100%) and a coated tongue was consistently noted in all children's age groups (71%–85% range). Signs and symptoms such as anaemia (71%), leukocytosis (47%), hepatitis (36%) and hepatomegaly (50%) were more common among pre–school children (under 5 years) than in other age groups, while headache and abdominal pain/tenderness was reported to be less common in this age group (14% and 20% respectively). Altered mental status (30%), signs of URTI (22%), leucopenia (57%), abdominal pain/tenderness (70%) were common in school–aged children. Headache (75%), abdominal distension (66%), cough (60%) and pneumonia (19%) were more common in older children aged 10–17 years. In contrast, more adults presented with nausea/vomiting (49%), splenomegaly (39%), GI perforation (5%), and thrombocytopenia (52%). Relative bradycardia, chills/rigors and dehydration were also frequently reported. Toxicity throughout the ages was found to be 26–38%. GI perforation was more common as age increased. Children were infected with MDR strains in 22–25% cases, as compared to more than half of enteric cases in adults which were MDR. Relapse was similar in all ages, but pre–schoolchildren had the highest fatality rates (6%), compared to all other age groups.

**Table 1:** Epidemiology of enteric fever by age (references to studies in **Online Supplementary Document(Online Supplementary Document)**)

	0–5 years (15 studies)*			5–10 years (8 studies)†			10–17 years (5 studies)‡			Adults ≥12 years (73 studies)§		
	**n**	**N**	**%**	**n**	**N**	**%**	**n**	**N**	**%**	**n**	**N**	**%**
**Signs and symptoms–systemic:**												
Fever	265	274	**97**	194	194	**100**	39	39	**100**	2287	2337	**98**
High grade fever	11	77	**14**							111	292	**38**
Headache	15	104	**14**	70	125	**56**	6	8	**75**	1149	1830	**63**
Toxicity	169	492	**34**	184	586	**31**	70	268	**26**	208	543	**38**
Rash or rose spots	2	12	**17**	6	93	**6**	5	84	**6**	75	982	**8**
Coated tongue	28	33	**85**	154	190	**81**	32	45	**71**	490	881	**56**
**Respiratory and abdominal:**												
Cough	58	187	**31**	78	231	**34**	32	53	**60**	595	1459	**41**
Nausea or vomiting	61	193	**32**	88	194	**45**	5	39	**13**	792	1628	**49**
Diarrhea	286	665	**43**	173	752	**23**	94	276	**34**	1118	2407	**46**
Constipation	52	439	**12**	177	752	**24**	31	276	**11**	382	1570	**24**
Hepatomegaly	261	526	**50**	276	630	**44**	93	245	**38**	555	1903	**29**
Splenomegaly	165	635	**26**	228	789	**29**	71	290	**24**	883	2278	**39**
Abdominal pain or tenderness	29	143	**20**	136	194	**70**	19	39	**49**	981	1827	**54**
Abdominal distention or ileus	24	131	**18**	90	190	**47**	35	53	**66**	139	831	**17**
**Laboratory features:**												
Anaemia (Hb <12 g/dl)	50	70	**71**	21	31	**68**				487	1687	**29**
Leukopenia (<5 × 10^3^/µL)	17	81	**21**	71	125	**57**				800	2248	**36**
Leukocytosis (>15 × 10^3^/µL)	198	417	**47**	91	558	**16**	27	237	**11**	32	238	**13**
**Complications:**												
Shock or hypotension	3	63	**5**	9	97	**9**	0	36	**0**	145	1559	**9**
Altered mental status	27	197	**14**	86	287	**30**	4	75	**5**	972	3339	**29**
Pneumonia or chest signs	28	194	**14**	57	318	**18**	15	81	**19**	205	1921	**11**
GI bleeding	8	158	**5**	11	189	**6**	6	75	**8**	177	2557	**7**
GI perforation	1	127	**1**	5	194	**3**	0	31	**0**	110	2183	**5**
**Outcome:**												
Relapse	18	399	**5**	29	614	**5**	14	273	**5**	66	1516	**4**
Death	38	656	**6**	10	751	**1**	1	75	**1**	197	4698	**4**

n – number with feature, N – number assessed

*Includes studies with data for under 1, under 2, under 3 and under 4 year–olds.

†Includes studies with data for 5 to 12, 5 to 13 and 6 to 12 year–olds.

‡Includes studies with data for 10 to 14, 10 to 15 and 10 to 17 year–olds.

§Includes a large number of adolescents, since author definition of adults was very varied (eg: 12 years and above, 15–59 years).

In comparing children 0–5 years with children aged 5–10 years, different features were found to be more likely to occur (Table S2 in **Online Supplementary Document(Online Supplementary Document)**), and after pooling data for children under 10 years compared to adults (author defined ages or aged 12 and above), the features more likely to occur in children are shown in and Table S3 in **Online Supplementary Document(Online Supplementary Document)**.

### Regional spectrum of enteric fever in children

Data was provided from above referenced studies on children as well as age disaggregated studies–in total 90 studies (**Table 2** and Tables S1 and S2 in **Online Supplementary Document(Online Supplementary Document)**). In almost all regions, 40% of enteric cases presented after receiving prior antibiotics. MDR enteric fever was highest in the Middle East & North Africa from 3 studies, followed by South Asia. Fluoroquinolone resistance was reported rarely in almost all regions. The most common feature globally was fever. Other common features were anaemia (highest in South Asia), hepatomegaly (commonest in East Asia & Pacific), and coated tongue. Toxicity and relative bradycardia was seen highest in Sub Saharan Africa. Diarrhea was more common than constipation, except in East Asia/Pacific. Sub Saharan Africa and Middle East/North Africa had a higher occurrence of abdominal distension and ileus, as well as GI perforation (6%). High income countries reported more weakness/malaise, rose spots and an intermittent pattern of fever. Relapse was consistently low: 2–9% and case–fatality rate ranged from 1–6%, highest in Sub Saharan Africa and North Africa/Middle East regions.

**Table 2:** Spectrum of enteric fever by economic and geographical regions (references to studies in **Online Supplementary Document(Online Supplementary Document)**)

	High income countries (17 studies)			Europe & Central Asia (4 studies)			Sub–Saharan Africa (12 studies)			Middle East & N. Africa (3 studies)			South Asia (41 studies)			East. Asia & Pacific (11 studies)		
	**N**	**N**	**%**	**n**	**N**	**%**	**n**	**N**	**%**	**n**	**N**	**%**	**N**	**N**	**%**	**n**	**N**	**%**
**Demographics/history:**																		
Blood/bone marrow isolates	540	710	**76**	122	131	**93**	1069	1382	**77**	250	281	**89**	5280	5736	**92**	891	1012	**88**
Pre–treatment antibiotics	10	13	**77**	6	80	**8**	15	35	**43**				1804	3152	**57**	255	570	**45**
Duration of illness ≤1 week	68	161	**42**				343	574	**60**	92	150	**61**	879	2013	**44**	203	325	**62**
Multi–drug resistant isolates	10	288	**3**	0	72	**0**	0	120	**0**	60	60	**100**	1885	4214	**45**	150	459	**33**
Fluoroquinolone resistance	0	12	**0**	0	8	**0**							25	1169	**2**	0	326	**0**
**Signs and symptoms–systemic:**																		
Fever	614	639	**96**	119	123	**97**	920	1005	**92**	103	131	**79**	4490	4800	**94**	673	688	**98**
High grade fever	3	13	**23**	72	72	**100**	204	438	**47**				2085	3178	**66**	68	126	**54**
Relative bradycardia	77	271	**28**	2	96	**2**	285	573	**50**	12	150	**8**	21	482	**4**	40	258	**16**
Headache	115	481	**24**	64	123	**52**	374	909	**41**	73	131	**56**	425	3730	**11**	209	609	**34**
Toxicity	1	71	**1**				198	378	**52**	8	71	**11**	1072	3486	**31**	83	231	**36**
Rash or rose spots	97	523	**19**	6	104	**6**	1	792	**0**	9	221	**4**	7	960	**1**	38	542	**7**
Dehydration	31	111	**28**	3	24	**13**	66	278	**24**	22	71	**31**	3	50	**6**	18	167	**11**
Coated tongue										81	150	**54**	314	608	**52**	140	195	**72**
**Respiratory and abdominal:**																		
Cough	122	434	**28**	23	96	**24**	146	426	**34**	149	281	**53**	530	2823	**19**	263	786	**33**
Nausea or vomiting	229	582	**39**	16	51	**31**	211	535	**39**	33	71	**46**	1634	4556	**36**	287	684	**42**
Diarrhea	269	609	**44**	26	51	**51**	592	1161	**51**	43	131	**33**	1335	4503	**30**	308	922	**33**
Constipation	73	383	**19**	3	24	**13**	61	324	**19**	0	0		254	3895	**7**	240	772	**31**
Hepatomegaly	145	414	**35**	79	123	**64**	103	405	**25**	62	131	**47**	2060	4510	**46**	545	801	**68**
Splenomegaly	168	512	**33**	55	123	**45**	153	555	**28**	195	281	**69**	1441	4714	**31**	239	989	**24**
Abdominal pain tenderness	215	555	**39**	20	51	**39**	420	975	**43**	77	131	**59**	978	3782	**26**	385	786	**49**
Abdominal distention or ileus	20	206	**10**	9	51	**18**	236	809	**29**	103	221	**47**	254	4097	**6**	198	732	**27**
**Laboratory features:**																		
Anaemia (Hb <12 g/dl)	78	209	**37**	11	25	**44**	410	936	**44**	16	62	**26**	2284	3132	**73**	191	566	**34**
Leukopenia (<5 × 10^3^/µL)	84	408	**21**	16	49	**33**	89	365	**24**	12	69	**17**	204	2069	**10**	213	653	**33**
Leukocytosis (>15 × 10^3^/µL)	1	41	**2**	3	49	**6**	14	113	**12**	11	69	**16**	384	1754	**22**	12	177	**7**
**Complications:**																		
Shock or hypotension	2	50	**4**				4	131	**3**	4	71	**6**	123	2606	**5**	10	278	**4**
Altered mental status	51	417	**12**	44	123	**36**	155	1127	**14**				414	4928	**8**	191	786	**24**
Pneumonia or chest signs	19	305	**6**	1	24	**4**	267	993	**27**	66	150	**44**	227	1966	**12**	66	785	**8**
GI bleeding	19	548	**3**	2	24	**8**	37	1069	**3**	1	150	**1**	82	1385	**6**	24	616	**4**
GI perforation	4	449	**1**				63	996	**6**	1	150	**1**	13	943	**1**	3	274	**1**
**Outcome:**																		
Relapse	34	560	**6**	4	58	**7**	34	938	**4**	18	210	**9**	194	3614	**5**	7	326	**2**
Death	5	567	**1**	5	131	**4**	79	1328	**6**	10	210	**5**	83	4981	**2**	7	922	**1**

n – number with feature, N – number assessed

In comparing Africa (Sub Saharan Africa and Middle East/ North Africa) to South Asia, significant findings more likely to occur in children from African countries are presented in Table S6 in **Online Supplementary Document(Online Supplementary Document)**.

### Hospital vs community–based studies in children

Data was derived from 83 prospective or retrospective studies or treatment trials of hospitalized, predominantly inpatient children from urban areas (**Table 3** and Table S7 in **Online Supplementary Document(Online Supplementary Document)**). Data meeting the pre–defined criteria was scarce other than from hospital based studies, and could be extracted from only 6 studies conducted in community settings or health centers (outpatient) on children. Hospitalized children (Table S8 in **Online Supplementary Document(Online Supplementary Document)**) presented most commonly with high–grade fever (odds ratio (OR) 4.7, 95% confidence interval (CI) 3.5–6.4), hepatomegaly (OR 7.1, 95% CI 4.2–12.0), nausea/vomiting (OR 2.0, 95% CI 1.6–2.6), abdominal distension (OR 7.4,95% CI 2.7–20.0), and coated tongue, anaemia and neutrophilia. Diarrhea (OR 5.2, 95% CI 3.8–7.2) and constipation (OR 4.2, 95% CI 1.9–9.6) were also associated more in hospitalized children. Other findings more likely to occur in hospitalized children were splenomegaly (OR 2.7, 95% CI 1.7–4.0); GI bleeding (OR 9.0, 95% CI 1.2–64.4); pre–treatment antibiotics received (OR 2.8, 95% CI 2.0–4.0) and duration of illness ≤1week (OR 1.8, 95% CI 1.3–2.4). Rose spots were an uncommon finding (5%). In all of the isolates reported in these studies, MDR was higher in hospitalized children compared to community based studies (OR 1.7, 95% CI 1.3–2.1).The most common complications in hospitalized children was DIC (18%), followed by pneumonia, arthritis/arthralgia, altered mental status, hepatitis, and meningitis (8–15%).

**Table 3:** Enteric fever in children in hospital–based vs community/health–center (references to studies in **Online Supplementary Document(Online Supplementary Document)**)

	Hospital–based (83 studies)			Community–based or health center (out–patients) (6 studies)		
	**n**	**N**	**%**	**n**	**N**	**%**
**Demographics/history:**						
Blood/bone marrow isolates	7693	8786	**88**	407	407	**100**
Pre–treatment antibiotics received	2047	3709	**55**	43	141	**30**
Duration of illness ≤1week	1520	3034	**50**	69	193	**36**
Multi–drug resistant isolates	2018	4856	**42**	93	313	**30**
Fluoroquinolone resistance	11	1433	**1**	9	44	**20**
**Signs and symptoms–systemic:**						
Fever	6327	6787	**93**	394	395	**100**
High grade fever	2372	3622	**65**	60	209	**29**
Bradycardia or relative bradycardia	437	1830	**24**			
Headache	1088	5577	**20**	169	368	**46**
Toxicity	1338	4171	**32**	18	28	**64**
Rash or rose spots	158	3142	**5**			
Dehydration	143	701	**20**			
Coated tongue	535	953	**56**			
**Respiratory and abdominal:**						
Cough	1153	4588	**25**	62	190	**33**
Nausea or vomiting	2300	6038	**38**	95	407	**23**
Diarrhea	2512	6886	**36**	42	423	**10**
Constipation	625	5209	**12**	6	193	**3**
Hepatomegaly	2946	6175	**48**	16	141	**11**
Splenomegaly	2193	6937	**32**	25	169	**15**
Abdominal pain or tenderness	1901	5873	**32**	186	369	**50**
Abdominal distention or ileus	814	5889	**14**	4	189	**2**
**Laboratory features:**						
Anaemia (Hb <12 g/dL)	2976	4908	**61**			
Leukopenia (<5 × 10^3^/µL)	602	3442	**17**	15	137	**11**
Leukocytosis (>15 × 10^3^/µL)	399	2033	**20**	17	136	**13**
**Complications:**						
Shock or hypotension	143	3136	**5**			
Altered mental status	853	7343	**12**			
Pneumonia or chest signs	647	4232	**15**			
GI bleeding	164	3603	**5**	1	189	**1**
GI perforation	84	2812	**3**			
**Outcome:**						
Relapse	291	5706	**5**			
Death	181	7909	**2**	0	209	**0**

n – number with feature, N – number assessed, Hb – hemoglobin, GI – gastrointestinal

### MDR vs sensitive isolates in children

Thirty six studies provided either disaggregated data for MDR and drug sensitive enteric fever or had all sensitive or all MDR isolates (**Table 4** and Table S9 in **Online Supplementary Document(Online Supplementary Document)**). Children infected with MDR isolates (sample size range from 11 to 1647) (Table S10 in **Online Supplementary Document(Online Supplementary Document)**) presented late (duration of illness >1week) (OR 2.7, 95% CI 2.1–3.4) with prior antibiotic treatment. Children infected with MDR strains were more toxic (OR 2.1, CI 1.6–2.6) and had relatively higher frequency of complications and adverse outcomes. Complications such as abdominal distention or ileus (OR 2.6, 95% CI 1.7–4.1), GI bleeding (OR 2.3, 95% CI 1.1–4.5), shock/hypotension (OR 2.9, 95% CI 1.2–7.3); myocarditis (OR 4.2, 95% CI 1.4–12.5) and pneumonia (OR 2.2, 95% CI 1.3–3.7) were higher in cases of MDR isolates compared to pan–sensitive isolates. High grade fever (OR 0.6, 95% CI 0.5–0.8), relapse (OR 0.3, 95% CI 0.1–0.7); leucopenia (OR 0.5, 95% CI 0.3–0.8); thrombocytopenia (OR 0.1, 95% CI 0.03–0.4) and arthritis or arthralgia/myalgia (OR 0.05,95% CI 0.01–0.4) were more frequent in children with sensitive isolates (sample size range from 13 to 2531). The case fatality was 1.0% vs 1.3% in resistant and sensitive enteric respectively.

**Table 4:** Demographic and clinical features of enteric fever in children with multi–drug resistant vs sensitive strains of *S. typhi* and *S. paratyphi* (references to studies in **Online Supplementary Document(Online Supplementary Document)**)

	Multi–drug resistant (14 studies)			Sensitive (22 studies)		
	**n**	**N**	**%**	**n**	**N**	**%**
**Demographics/history:**						
Multi–drug resistant isolates	1647	1647	**100**	0	2531	**0**
Chloramphenicol resistance	125	125	**100**	9	293	**3**
Fluoroquinolone resistance	0	183	**0**	5	55	**9**
Blood/bone marrow Isolates	1121	1121	**100**	1600	1616	**99**
Pre–treatment antibiotics received	10	11	**91**	71	335	**21**
Duration of illness ≤1week	151	417	**36**	627	1034	**61**
**Signs and symptoms–systemic:**						
Fever	620	653	**95**	1350	1393	**97**
High grade fever	441	552	**80**	945	1090	**87**
Bradycardia or relative bradycardia	7	62	**11**	3	218	**1**
Headache	116	220	**53**	122	382	**32**
Toxicity	208	427	**49**	316	1006	**31**
Rash or rose spots	3	153	**2**	11	292	**4**
Dehydration	6	15	**40**	11	190	**6**
Coated tongue	34	77	**44**	8	13	**62**
**Respiratory and abdominal:**						
Cough	92	256	**36**	151	487	**31**
Nausea or vomiting	140	332	**42**	128	371	**35**
Diarrhea	201	638	**32**	464	1358	**34**
Constipation	52	487	**11**	137	1203	**11**
Hepatomegaly	456	725	**63**	699	1639	**43**
Splenomegaly	320	694	**46**	433	1669	**26**
Abdominal pain or tenderness	294	600	**49**	358	1259	**28**
Abdominal distention or ileus	55	260	**21**	40	434	**9**
**Laboratory features:**						
Anaemia (Hb <12 g/dL)	296	442	**67**	656	1170	**56**
Leukopenia (<5 × 10^3^/µL)	20	362	**6**	132	1177	**11**
Leukocytosis (>15 × 10^3^/µL)	80	300	**27**	231	914	**25**
**Complications:**						
Shock or hypotension	20	115	**17**	7	105	**7**
Altered mental status	80	586	**14**	83	638	**13**
Pneumonia or chest signs	32	219	**15**	35	484	**7**
GI bleeding	28	382	**7**	12	357	**3**
GI perforation	4	142	**3**	1	136	**1**
**Outcome:**						
Relapse	7	329	**2**	12	151	**8**
Death	23	1647	**1.0**	31	2339	**1.3**

n – number with feature, N – number assessed

Other significant features more likely to be seen in MDR cases are shown in Table S10 in **Online Supplementary Document(Online Supplementary Document)**.

## DISCUSSION

Despite advances in public health and hygiene that have led to a disappearance of enteric fever from much of the developed world, it still remains the commonest bacteraemic illness in South Asian countries with children being especially susceptible [1,14]. The emergence of multi–drug resistance is very concerning due to the limited therapeutic options, high financial implications and its continuing burden in impoverished, low–income countries [6,14,21].

Several limitations should be recognized in considering our data. Our inclusion of culture proven and serological confirmed cases with culture confirmation in at least 50% of these cases may not reflect the true clinical features profile of enteric fever. With the high prevalence of prior antibiotic treatment, culture proven diagnosis may have been falsely low. On the contrary, exclusion of clinically diagnosed cases may also have resulted in missing out enteric fever with atypical features. An overwhelming majority of included studies were from urban areas, with many studies from rural areas excluded for reasons such as mixed reporting of adults and children, or diagnosis solely on clinical features (**Box 1**). Many community level studies were also excluded due to similar reasons. Existing literature gives a varying, non–standardized representation of enteric fever since there are differences in definitions such as adult/pediatric age group cutoffs, relapse, altered mental status and other clinical features. Case series (such as “all complicated cases excluded”, or “all with diarrhea”) were excluded as well. Paratyphoid fever was not included to be reviewed in this systematic review as it has a different clinical spectrum, however in 28% of cases data could not be separated from typhoid fever.

Outcomes such as resistance, relapse, and mortality were not reported in all studies, leading to an incomplete representation. Confounders, such as co–morbidities, resistance, socio–economic status, heterogeneous access to health could not be adjusted for since individual level data were not analyzed. Current trends in resistance especially nalidixic acid resistance and emerging fluoroquinolone resistance have not been extensively reported. Most studies were from South Asia, especially India and Pakistan. Regions were categorized based on the World Bank list of economies, which gives geographic classifications for low–income and middle–income economies only, while high income countries that may reflect any geographical region with an improved developmental status. Furthermore, our review is not fully representative of non–English language speaking regions of the world, although data from translated abstracts were used where possible.

Notwithstanding the above, our review highlights a number of key findings of the epidemiological pattern of enteric fever in different categories, which will assist the clinician in his diagnosis and help in the fight against enteric fever. Most of our data are from urban, hospitalized children who were more likely to have the following features: high–grade fever, nausea/vomiting, diarrhea, constipation, hepatomegaly, splenomegaly, neutrophilia, abdominal distension and GI bleeding. Young children (under 5 years) were more likely to show anaemia, diarrhea, leukocytosis, hepatitis and hepatomegaly and had a higher mortality. Older children commonly showed an altered mental status, signs of URTI leucopenia, and abdominal pain/tenderness. Adults were more likely to present with splenomegaly, GI perforation, and thrombocytopenia.

In previous literature, the commonest complications are reported to be gastrointestinal bleeding, intestinal perforation, encephalopathy and shock [5,14,33], though our review suggests that DIC, pneumonia, arthritis/arthralgia altered mental status, hepatitis, and meningitis predominate. The high frequency of DIC in our review was determined from 4 studies with one study forming the majority of the data [17]. Of note, the ‘classic’ stepladder temperature pattern [34] was only present in 25% of adult patients. Amongst children in the preschool years, a high case–fatality rate of 6% was found from the included studies, and death was 4.5 times more likely to occur compared to school–aged children. One included study had a particularly strong association of mortality in younger children with anaemia [17]. This high mortality and high incidence [25,35] identifies this age group as a high risk group to be addressed for vaccinations.

Other related or underlying factors influencing the clinical profile and outcomes of enteric fever are varying strain virulence, inoculum size, delays in or duration of treatment received, numerous host factors such as immune response, co–existing illnesses or infections, or underlying malnutrition [5,12,36]. These findings must be considered with caution, as our review was limited to studies with full reporting of clinical features and many studies with only outcome data were excluded. Others have recently reported increased disease severity with emergence of fluoroquinolone resistance [37].

There is insufficient and inconsistent reporting of clinical features data in MDR isolates, especially in the 1980s when the first few outbreaks were reported [5]. This may be due to a publication bias, since chloramphenicol resistance data was being reported at 10% from that time period [5].The complications are higher with multi–drug resistant strains and these isolates have been shown to be more virulent than sensitive strains [38,39].In this review, the case–fatality rates from all resistant and all sensitive *S. typhi* were almost the same (1.0% in MDR strains vs 1.3% in sensitive strains), reflecting a general decrease in overall mortality in treated cases since the advent of antibiotic usage and improved health care, as our review is mostly derived from inpatient reports (77% of studies).

The case fatality rate of 2% from 83 studies in hospitalized children, is comparable to case–fatality rates reviewed by Crump et al. [18] from 10 population–based studies (although in mixed age groups) which showed a range of 0–1.8%. However, regionally, Sub–Saharan Africa, and North Africa and Middle East had the highest case–fatality rates (5–6%). The relapse rate was low, ranging from 2–9% in all regions, reflecting improved hospital care and initiation of antibiotics, while regional differences in case–fatality rate ranged from 1–6%, highest in Sub Saharan Africa and North Africa/Middle East regions. This may reflect the higher rate of complications such as GI perforation, GI bleed and pneumonia in these regions. As this data spans studies prior to the onset of improved health care access and surgical treatments, as well as after it reflects the overall picture of mortality enteric fever has posed on each region.

Widespread antibiotic pre–treatment was present in all regions, except Europe and Central Asia, due to prevalence of self–medication and poor health–seeking behaviors [40]. This has implications for the development of newer diagnostic tests that can replace blood culture, and ideally be more rapid, specific and cost-effective as well as sensitive. Rational use of antibiotics based on culture sensitivity patterns in different regions in imperative in curtailing the further evolution of multi–drug resistance which is already rife.

### Applicability and implications for research

Although enteric fever is essentially a pediatric disease in South Asia, there is a serious dearth of data from children in community settings in global literature [3,23,25,41-43]. Hospital–based data helps show severity of infection and outcomes associated with treatment, but capturing data on clinical features from studies based in the community is imperative to strengthen our ability to pick and treat enteric fever in the most vulnerable and to better understand presentation of drug resistance and treatment outcomes of mild enteric fever. Treatment requires a low threshold for empirical antibiotics but this must be weighed against the growing rates of resistance in many regions that make treatment options complex and costly. The solution will have to be multi–faceted and include improved sanitation, vaccination implementation in high–risk populations in combination with rapid diagnosis, elimination of carriers, and rational use of the antibiotic options. Vaccinations as part of national immunizations programs (EPI) for those under 2 years of age in high risk populations will have to be the key in restriction of the spread of disease through reducing both disease transmission and new carriers, until water and sanitation are universally upgraded [33,44,45].

Future studies should be designed keeping these gaps in mind and focus on community based enteric cases. Descriptions of all clinical features, resistance patterns and mortality should be a primary objective of researchers in treatment trials, vaccine trials and prospective/retrospective studies, preferably in separate cohorts based on age (children vs adults), using standardized, clearly defined age categories. The cut–offs for MIC for fluoroquinolones have been recently revised and reports should include references of the MIC used by their laboratory. There is a need for randomized control trials for appropriate outpatient therapy in the face of rising resistance to commonly used antimicrobials.

### Surveillance networks

There is a need to establish a consortium for reporting of enteric fever, especially with regard to AMR (antimicrobial resistance) as well as a central repository for genomic studies, looking at SNP related to enteric severity. The Coalition Against Typhoid [46] for example, is a global forum of health and immunization experts working to expedite and sustain evidence–based decisions at the global, regional and national levels regarding the use of enteric vaccination to prevent childhood enteric fever. They state the need to develop long and short term goals for enteric control, which include for the short term high burden and at risk populations immunizations, good hygiene practices, and for long term improvements in access to safe water and improved sanitation as their goals.

## References

[1] Crump JA, Luby SP (2004). Mintz ed. The global burden of typhoid fever.. Bull World Health Organ.

[2] Crump JA, Mintz ED (2010). Global trends in typhoid and paratyphoid fever.. Clin Infect Dis.

[3] Ochiai RL, Acosta CJ, Danovaro–Holliday MC, Baiqing D, Bhattacharya SK, Agtini MD (2008). A study of typhoid fever in five Asian countries: disease burden and implications for controls.. Bull World Health Organ.

[4] Aghanwa HS, Morakinyo O (2001). Correlates of psychiatric morbidity in typhoid fever in a Nigerian general hospital setting.. Gen Hosp Psychiatry.

[5] Bhan MK, Bahl R, Bhatnagar S (2005). Typhoid and paratyphoid fever.. Lancet.

[6] Bhutta ZA (2006). Current concepts in the diagnosis and treatment of typhoid fever.. BMJ.

[7] Breiman RF, Cosmas L, Njuguna H, Audi A, Olack B, Ochieng JB (2012). Population–based incidence of typhoid fever in an urban informal settlement and a rural area in Kenya: implications for typhoid vaccine use in Africa.. PLoS ONE.

[8] Catalano G, Del Vecchio–Blanco C, Varone GL (1972). Changing pattern of typhoid fever: clinical bacteriological and immunological aspects.. Boll Ist Sieroter Milan.

[9] Dutta TK (2001). Beeresha, Ghotekar LH. Atypical manifestations of typhoid fever.. J Postgrad Med.

[10] Vollaard AM, Ali S, Widjaja S, Asten HA, Visser LG, Surjadi C (2005). Identification of typhoid fever and paratyphoid fever cases at presentation in outpatient clinics in Jakarta, Indonesia.. Trans R Soc Trop Med Hyg.

[11] Hosoglu S, Geyik MF, Akalin S, Ayaz C, Kokoglu OF, Loeb M (2006). A simple validated prediction rule to diagnose typhoid fever in Turkey.. Trans R Soc Trop Med Hyg.

[12] Parry CM, Hien TT, Dougan G, White NJ, Farrar JJ (2002). Typhoid fever.. N Engl J Med.

[13] Siddiqui FJ, Rabbani F, Hasan R, Nizami SQ, Bhutta ZA (2006). Typhoid fever in children: some epidemiological considerations from Karachi, Pakistan.. Int J Infect Dis.

[14] World Health Organization; Typhoid Immunization Working Group. Background paper on vaccination against typhoid fever using new–generation vaccines. Presented at the SAGE November 2007 meeting. Available: http://www.who.int/immunization/SAGE_Background_publicpaper_typhoid_newVaccines.pdf. Accessed: 7 September 2014.

[15] Chau TT, Campbell JI, Galindo CM, Hoang NVM, Diep TS, Nga TTT (2007). Antimicrobial drug resistance of Salmonella enterica serovar Typhi in Asia and molecular mechanism of reduced susceptibility to the fluoroquinolones.. Antimicrob Agents Chemother.

[16] Kumar R, Gupta N (2007). Multidrug-resistant typhoid fever.. Indian J Pediatr.

[17] Bhutta ZA (1996). Impact of age and drug resistance on mortality in typhoid fever.. Arch Dis Child.

[18] Crump JA, Ram PK, Gupta SK, Miller MA, Mintz ED, Part I (2008). Analysis of data gaps pertaining to Salmonella enterica serotype Typhi infections in low and medium human development index countries, 1984–2005.. Epidemiol Infect.

[19] Walia M, Gaind R, Paul P, Mehta R, Aggarwal P, Kalaivani M (2006). Age–related clinical and microbiological characteristics of enteric fever in India.. Trans R Soc Trop Med Hyg.

[20] Bhutta ZA, Naqvi SH, Razzaq RA, Farooqui BJ (1991). Multidrug–resistant typhoid in children: presentation and clinical features.. Rev Infect Dis.

[21] Ochiai RL, Acosta CJ, Agtini M, Bhattacharya SK, Bhutta ZA, Do CG (2007). The use of typhoid vaccines in Asia: the DOMI experience.. Clin Infect Dis.

[22] Thaver D, Zaidi AK, Critchley J, Madni SA, Bhutta ZA (2005). eFluoroquinolones for treating typhoid and paratyphoid fever (enteric fever).. Cochrane Database Syst. Rev.

[23] Brooks WA, Hossain A, Goswami D, Nahar K, Alam K, Ahmed N (2005). Bacteremic typhoid fever in children in an urban slum, Bangladesh.. Emerg Infect Dis.

[24] Butler T, Islam A, Kabir I, Jones PK (1991). Patterns of morbidity and mortality in typhoid fever dependent on age and gender: review of 552 hospitalized patients with diarrhea.. Rev Infect Dis.

[25] Sinha A, Sazawal S, Kumar R, Sood S, Reddaiah VP, Singh B (1999). Typhoid fever in children aged less than 5 years.. Lancet.

[26] Ferreccio C, Levine MM, Manterola A, Rodriguez G, Rivara I, Prenzel I (1984). Benign bacteremia caused by Salmonella typhi and paratyphi in children younger than 2 years.. J Pediatr.

[27] Topley JM (1986). Mild typhoid fever.. Arch Dis Child.

[28] Sen SK, Mahakur AC (1972). Enteric fever–a comparative study of adult and paediatric cases.. Indian J Pediatr.

[29] Worku B (2000). Typhoid fever in an Ethiopian children's hospital: 1984–1995.. Ethiop J Health Dev..

[30] Chiu CH, Tsai JR, Ou JT, Lin TY (2000). Typhoid fever in children: a fourteen–year experience.. Acta Paediatr Taiwan.

[31] World Bank. World Bank list of economies. Available: http://siteresources.worldbank.org/DATASTATISTICS/Resources/CLASS.XLS. Accessed: 1 April 2012.

[32] Dean AG, Sullivan KM, Soe MM. OpenEpi: Open Source Epidemiologic Statistics for Public Health, Version updated 2015/05/04. Available: www.OpenEpi.com. Accessed: 22 June 2011.

[33] World Health Organization. Department of Vaccines and Biologicals. Background document: the diagnosis, prevention and treatment of typhoid fever. Geneva: WHO, 2003. Available at: www.who.int/entity/vaccine_research/documents/en/typhoid_diagnosis.pdf. Accessed: 25 August 2015.

[34] Mandell GL, Bennett JE, Dolin R. Mandell, Douglas, and Bennett's principles and practice of infectious disease. 7th ed. Philadelphia: Churchill Livingstone Elsevier; 2010.

[35] Podda A, Saul AJ, Arora R, Bhutta Z, Sinha A, Gaind R (2010). Conjugate vaccines for enteric fever: proceedings of a meeting organized in New Delhi, India in 2009.. J Infect Dev Ctries.

[36] Bhutta ZA (1996). Impact of age and drug resistance on mortality in typhoid fever.. Arch Dis Child.

[37] Parry CM, Thompson C, Vinh H, Chinh NT, Ho VA, Hien TT (2014). Risk factors for the development of severe typhoid fever in Vietnam.. BMC Infect Dis.

[38] Nesbitt A, Mirza NB (1989). Salmonella septicaemias in Kenyan children.. J Trop Pediatr.

[39] Thong KL, Passey M, Clegg A, Combs BG, Yassin RM, Pang T (1996). Molecular analysis of isolates of Salmonella typhi obtained from patients with fatal and nonfatal typhoid fever.. J Clin Microbiol.

[40] Bhutta ZA (2006). Current concepts in the diagnosis and treatment of typhoid fever.. BMJ.

[41] Lin FY, Vo AH, Phan VB, Nguyen TT, Bryla D, Tran CT (2000). The epidemiology of typhoid fever in the Dong Thap Province, Mekong Delta region of Vietnam.. Am J Trop Med Hyg.

[42] Owais A, Sultana S, Zaman U, Rizvi A, Zaidi AKM (2010). Incidence of typhoid bacteremia in infants and young children in southern coastal Pakistan.. Pediatr Infect Dis J.

[43] Saha SK, Baqui AH, Hanif M, Darmstadt GL, Ruhulamin M, Nagatake T (2001). Typhoid fever in Bangladesh: implications for vaccination policy.. Pediatr Infect Dis J.

[44] Bhutta ZA, Capeding MR, Bavdekar A, Marchetti E, Ariff S, Soofi SB (2014). Immunogenicity and safety of the Vi–CRM 197 conjugate vaccine against typhoid fever in adults, children, and infants in south and southeast Asia: results from two randomised, observer–blind, age de–escalation, phase 2 trials.. Lancet Infect Dis.

[45] Park SE, Marks F (2014). A conjugate vaccine against typhoid fever.. Lancet Infect Dis.

[46] Coalition Aagainst Typhoid. Coalition against typhoid 2015. Available: http://www.coalitionagainsttyphoid.org/. Accessed: 4 September 2014.

