# Supplementary material for: Systematic review of the global epidemiology, clinical and laboratory profile of enteric fever
Source: J Glob Health. 2015 Nov 24;5(2):020407. doi: 10.7189/jogh.05.020407 (PMC4672836; doi:10.7189/jogh.05.020407)
Supplement: Online Supplementary Document [file jogh-05-020407-s001.pdf]

Online Supplementary Document

Azmatullah et al. Systematic review of the global epidemiology, clinical and laboratory profile of enteric fever

J Glob Health 2015;5:010407

Supplementary tables and definitions of variables

- Table S1: Epidemiology of enteric fever by age

	**0 to 5 years** 14 studies			**5 to 10 years** 6 studies			**10 to 17 years** 4 studies			**Adults (12 years & up)** 69 studies		
**Demographics/History**	**N**	**N**	**%**	**n**	**N**	**%**	**n**	**N**	**%**	**n**	**N**	**%**
Males	314	529	**59**	421	714	**59**	135	237	**57**	2761	4551	**61**
Blood/bone marrow Isolates	615	655	**94**	695	695	**100**	282	282	**100**	4153	4465	**93**
Pre-treatment antibiotics received	2	16	**13**							510	1240	**41**
Duration of illness ≤ 1week	13	22	**59**							287	506	**57**
Duration of illness within 1-2weeks	7	18	**39**							45	195	**23**
Duration of illness > 2 weeks	2	18	**11**							115	691	**17**
Duration of illness > 1 week	9	18	**50**							293	971	**30**
Multi-drug resistant isolates	94	383	**25**	121	558	**22**	58	237	**24**	913	1726	**53**
Fluoroquinolone resistance	10	161	**6**							11	1662	**1**
Chloramphenicol resistance	47	99	**47**							1119	1877	**60**
Ampicillin/Amoxycillin resistance	36	71	**51**							1081	1461	**74**
Co-trimoxazole resistance	28	71	**39**							936	1158	**81**
**Laboratory features**												
Severe anaemia (<7.5 g/dl)	45	91	**49**							17	437	**4**
Neutrophilia(>7.5 x 10^3/µL)	9	9	**100**							4	50	**8**
Thrombocytopenia (<150 x 10^3/µL)	0	12	**0**	5	28	**18**	4	31	**13**	210	395	**53**
Severe thrombocytopenia(<50 x 10^3/µL)										13	194	**7**
Hemolysis or hemolytic anaemia										1	100	**1**
**Other signs and symptoms**												
Chills or rigors	4	33	**12**	4	125	**3**				362	769	**47**
Bradycardia/relative bradycardia										532	1142	**47**
Dehydration							1	8	**13**	164	288	**57**
Anorexia/refusal to feed	36	55	**65**	82	125	**66**				355	765	**46**
Weakness/dizziness or malaise							5	8	**63**	223	472	**47**
Continuous fever	8	18	**44**							113	274	**41**
Intermittent fever	5	18	**28**							32	191	**17**
Remittent fever	5	18	**28**							86	199	**43**
Stepladder fever										63	255	**25**
Chelosis										2	82	**2**
URTI	5	34	**15**	14	65	**22**	5	45	**11**	34	334	**10**
**Other complications**												
Myocarditis	1	51	**2**	6	125	**5**				72	862	**8**
Meningitis or meningismus	7	139	**5**	3	153	**2**	0	31	**0**	29	1645	**2**
Hepatitis or raised LFTs	17	47	**36**	19	72	**26**				547	1955	**28**
Jaundice	6	46	**13**	0	28	**0**	1	39	**3**	43	985	**4**
DIC										22	573	**4**
Acute acalculouscholecystitis	7	71	**10**							7	97	**7**
Edema or pleural effusion or ascites	0	12	**0**	6	69	**9**	1	31	**3**	9	512	**2**
Convulsions	10	122	**8**	3	125	**2**	0	8	**0**			

Focal neurological involvement										96	1102	**9**
Epistaxis/bleeding (unspecified)	16	140	**11**	16	166	**10**				15	315	**5**
Any abscess	1	71	**1**							16	435	**4**
Osteomyelitis or septic arthritis	11	116	**9**							2	30	**7**
Renal involvement	2	71	**3**							140	1277	**11**
Arthritis or arthralgia or myalgia	0	18	**0**	1	28	**4**	4	39	**10**	194	1285	**15**

- **Table S2: Associations of clinical features in enteric fever by age (0-5 yrs old versus 5-10 yrs old)**

Clinical Features	Odds Ratio	95% Confidence Interval
Headache	0.1	0.05 - 0.2
Diarrhea	2.5	2.0 - 3.2
Constipation	0.4	0.3 - 0.6
Nausea or vomiting	0.6	0.4 - 0.8
Hepatomegaly	1.3	1.0 - 1.6
Abdominal pain or tenderness	0.1	0.07 - 0.2
Abdominal distention or ileus	0.2	0.1 - 0.4
Leucopenia (< 5 x 10^3/µL)	0.2	0.1 - 0.4
Leucocytosis(> 15 x 10^3/µL)	4.6	3.5 - 6.2
Altered mental status	0.4	0.2 - 0.6
Chills or rigors	4.1	1.0 - 17.7
Death	4.6	2.3 - 9.2

- **Table S3: Associations of clinical features in enteric fever by age (children versus adults[*])**

Clinical Features	Odds Ratio	95% Confidence Interval
Multi-drug resistant isolates	0.3	0.2- 0.3
Pre-treatment antibiotics received	0.2	0.04 - 0.9
Coated tongue	3.5	2.5- 5.1
Cough	0.7	0.6- 0.9
Nausea or vomiting	0.7	0.5- 0.8
Diarrhea	0.6	0.5 - 0.6
Constipation	0.7	0.6 - 0.9
Hepatomegaly	2.1	1.8- 2.5
Splenomegaly	0.6	0.5- 0.7
Abdominal distention or ileus	2.7	2.0- 3.7
Anemia (Hb < 12 g/dl)	5.8	3.8- 9.0
Leucopenia (< 5 x 10^3/µL)	1.4	1.0- 1.8
Leucocytosis (> 15 x 10^3/µL)	2.7	1.8- 4.0
Thrombocytopenia (<150 x 10^3/µL)	0.1	0.05- 0.3
Altered mental status	0.7	0.6- 0.9
GI perforation	0.4	0.2- 0.8
Pneumonia or chest signs	1.7	1.3- 2.2
Hepatitis or raised LFTs	5.9	3.9 - 8.8
Jaundice	1.9	1.0 - 3.5
Chills or rigors	0.06	0.03- 0.1

URTI	2.1	1.1- 3.9
High grade fever	0.3	0.1 - 0.5
Fluoroquinolone resistance	9.4	3.9- 22.5
Chloramphenicol resistance	0.6	0.4 - 0.9
Ampicillin/Amoxycillin resistance	0.4	0.2 - 0.6
Co-trimoxazole resistance	0.2	0.1 - 0.3
Severe anaemia (<7.5)	24.2	12.8 - 45.6
Anorexia/refusal to feed	2.2	1.6 - 3.1
Edema or pleural effusion or ascites	4.5	1.5 - 12.9
Epistaxis/bleeding (unspecified)	2.3	1.2 - 4.4
Renal involvement	0.2	0.06 - 1.0
Arthritis or arthralgia or myalgia	0.1	0.02 - 1.0

* Children - 0 - 5 yrs old versus 5 - 10 yrs old
Adults - Author defined or aged 12 years and above

References to Table 1 , Table S1, S2 and S3: Epidemiology of enteric fever by age

<u>Ages 0- 5 years</u>

1. Bhutta ZA. Impact of age and drug resistance on mortality in typhoid fever. ArchDisChild. 1996;75(3):214-7.
2. Chiu CH, Lin TY. Typhoid fever in children. Lancet. 1999;354(9194):2001-2.
3. Chiu CH, Tsai JR, Ou JT, Lin TY. Typhoid fever in children: a fourteen-year experience. Acta PaediatrTaiwan. 2000;41(1):28-32.
4. Duggan MB, Beyer L. Enteric fever in young Yoruba children. ArchDisChild. 1975;50(1):67-71.
5. Emary K, Moore CE, Chanpheaktra N, An KP, Chheng K, Sona S, et al. Enteric fever in Cambodian children is dominated by multidrug-resistant H58 Salmonella enterica serovar Typhi with intermediate susceptibility to ciprofloxacin. Trans R Soc Trop Med Hyg. 2012 Dec;106(12):718-24.
6. Ferreccio C, Levine MM, Manterola A, Rodriguez G, Rivara I, Prenzel I, et al. Benign bacteremia caused by Salmonella typhi and paratyphi in children younger than 2 years. J Pediatr. 1984;104(6):899-901.
7. Garg RA, Krashak R. Typhoid fever before two years of age. Indian Pediatr. 1993;30(6):805-8.
8. Green SD, Cheesbrough JS. Salmonella bacteraemia among young children at a rural hospital in western Zaire. AnnTropPaediatr. 1993;13(1):45-53.
9. Oh HM, Masayu Z, Chew SK. Typhoid fever in hospitalized children in Singapore. J Infect. 1997;34(3):237-42.
10. Owais A, Sultana S, Zaman U, Rizvi A, Zaidi AK. Incidence of typhoid bacteremia in infants and young children in southern coastal Pakistan. Pediatr Infect Dis J. 2010 Nov;29(11):1035-9.
11. Palacios Malmaceda PG, Vela Acosta JJ, Gutierez AW. [Typhoid fever in children under 2 years of age]. BolMedHospInfant Mex. 1981;38(3):473-83.
12. Rajajee S, Anandi TB, Subha S, Vatsala BR. Patterns of resistant Salmonella typhi infection in infants. JTropPediatr. 1995;41(1):52-4.

13. Sinha A, Sazawal S, Kumar R, Sood S, Reddaiah VP, Singh B, et al. Typhoid fever in children aged less than 5 years. Lancet. 1999;354(9180):734-7.
14. Soelistyowati S, Soenarto Y, Soesilo H, Widiarto, Widiatmodjo, Ismangoen. Typhoid fever in children. PaediatrIndones. 1982;22(7-8):138-46.
15. Walia M, Gaind R, Paul P, Mehta R, Aggarwal P, Kalaivani M. Age-related clinical and microbiological characteristics of enteric fever in India. TransRSocTropMed Hyg. 2006;100(10):942-8.
16. Wang JL, Kao JH, Tseng SP, Teng LJ, Ho SW, Hsueh PR. Typhoid fever and typhoid hepatitis in Taiwan. EpidemiolInfect. 2005;133(6):1073-9.

Ages 5 -10 years
1. Bhutta ZA. Impact of age and drug resistance on mortality in typhoid fever. ArchDisChild. 1996;75(3):214-7.
2. Chiu CH, Lin TY. Typhoid fever in children. Lancet. 1999;354(9194):2001-2.
3. Chiu CH, Tsai JR, Ou JT, Lin TY. Typhoid fever in children: a fourteen-year experience. Acta PaediatrTaiwan. 2000;41(1):28-32.
4. Emary K, Moore CE, Chanpheaktra N, An KP, Chheng K, Sona S, et al. Enteric fever in Cambodian children is dominated by multidrug-resistant H58 Salmonella enterica serovar Typhi with intermediate susceptibility to ciprofloxacin. Trans R Soc Trop Med Hyg. 2012 Dec;106(12):718-24
5. Leung DT, Bogetz J, Itoh M, Ganapathi L, Pietroni MA, Ryan ET, et al. Factors associated with encephalopathy in patients with Salmonella enterica serotype Typhi bacteremia presenting to a diarrheal hospital in Dhaka, Bangladesh. Am J Trop Med Hyg. 2012 Apr;86(4):698-702.
6. Moussa K. Enterica of childhood. J EgyptPublic Health Assoc. 1970;45(1):48-55.
7. Oh HM, Masayu Z, Chew SK. Typhoid fever in hospitalized children in Singapore. J Infect. 1997;34(3):237-42.
8. Soelistyowati S, Soenarto Y, Soesilo H, Widiarto, Widiatmodjo, Ismangoen. Typhoid fever in children. PaediatrIndones. 1982;22(7-8):138-46.
9. Walia M, Gaind R, Paul P, Mehta R, Aggarwal P, Kalaivani M. Age-related clinical and microbiological characteristics of enteric fever in India. TransRSocTropMed Hyg. 2006;100(10):942-8.

Ages 10-17yrs
1. Bhutta ZA. Impact of age and drug resistance on mortality in typhoid fever. ArchDisChild. 1996;75(3):214-7.
2. Chiu CH, Lin TY. Typhoid fever in children. Lancet. 1999;354(9194):2001-2.
3. Chiu CH, Tsai JR, Ou JT, Lin TY. Typhoid fever in children: a fourteen-year experience. Acta PaediatrTaiwan. 2000;41(1):28-32.
4. Emary K, Moore CE, Chanpheaktra N, An KP, Chheng K, Sona S, et al. Enteric fever in Cambodian children is dominated by multidrug-resistant H58 Salmonella enterica serovar Typhi with intermediate susceptibility to ciprofloxacin. Trans R Soc Trop Med Hyg. 2012 Dec;106(12):718-24
5. Kanra G, Yurdakok M, Secmeer G, Arvas A, Dereli O, Sarikayalar F, et al. Clinical findings of pediatric patients during an outbreak of typhoid fever in Ankara. TurkJ Pediatr. 1985;27(1):11-5.
6. Moussa K. Enterica of childhood. J EgyptPublic Health Assoc. 1970;45(1):48-55.

Adults
1. Abraham G, Teklu B. Typhoid fever: clinical analysis of 50 Ethiopian patients. EthiopMed J. 1981;19(2):41-6.
2. Aghanwa HS, Morakinyo O. Correlates of psychiatric morbidity in typhoid fever in a Nigerian general hospital setting. GenHospPsychiatry. 2001;23(3):158-62.
3. Ali G, Rashid S, Kamli MA, Shah PA, Allaqaband GQ. Spectrum of neuropsychiatric complications in 791 cases of typhoid fever. TropMed Int Health. 1997;2(4):314-8.
4. Abdool Gaffar MS, Seedat YK, Coovadia YM, Khan Q. The white cell count in typhoid fever. TropGeogrMed. 1992;44(1-2):23-7.
5. Alsoub H, Uwaydah AK, Matar I, Zebeib M, Elhag KM. A clinical comparison of typhoid fever caused by susceptible and multidrug-resistant strains of Salmonella typhi. BrJ ClinPract. 1997;51(1):8-10.
6. Alam MN, Haq SA, Das KK, Baral PK, Mazid MN, Siddique RU, et al. Efficacy of ciprofloxacin in enteric fever: comparison of treatment duration in sensitive and multidrug-resistant Salmonella. AmJTropMedHyg. 1995;53(3):306-11.
7. Barrett FC, Knudsen JD, Johansen IS. Cases of typhoid fever in Copenhagen region: a retrospective study of presentation and relapse. BMC research notes. 2013;6:315.
8. Bhatia RS. Changing trends in the presentation and treatment of enteric fever: our experience. The Journal of the Association of Physicians of India. 1992;40(4):283-4.
9. Butler T, Bell WR, Levin J, Linh NN, Arnold K. Typhoid fever. Studies of blood coagulation, bacteremia, and endotoxemia. Arch Intern Med. 1978 Mar;138(3):407-10.
10. Butler T, Linh NN, Arnold K, Pollack M. Chloramphenicol-resistant typhoid fever in Vietnam associated with R factor. Lancet. 1973;2(7836):983-5.
11. Carcelen A, Chirinos J, Yi A. Furazolidone and chloramphenicol for treatment of typhoid fever. ScandJGastroenterolSuppl. 1989;169:19-23.
12. Catalano G, Del Vecchio-Blanco C, Varone GL. Changing pattern of typhoid fever: clinical bacteriological and immunological aspects. BollIstSieroterMilan. 1972;51(2):157-64.
13. Caumes E, Ehya N, Nguyen J, Bricaire F. Typhoid and paratyphoid fever: a 10-year retrospective study of 41 cases in a Parisian hospital. J TravelMed. 2001;8(6):293-7.
14. Chakravorty B, Jain N, Gupta B, Rajvanshi P, Sen MK, Krishna A. Chloramphenicol resistant enteric fever. J Indian Med Assoc. 1993;91(1):10-3.
15. Clark TW, Daneshvar C, Pareek M, Perera N, Stephenson I. Enteric fever in a UK regional infectious diseases unit: a 10 year retrospective review. J Infect. 2010 Feb;60(2):91-8.
16. Chuttani HK, Jain K, Misra RC. Small bowel in typhoid fever. Gut. 1971 Sep;12(9):709-12.
17. Daga MK, Sarin K, Sarkar R. A study of culture positive multidrug resistant enteric fever--changing pattern and emerging resistance to ciprofloxacin. J AssocPhysicians India. 1994;42(8):599-600.
18. Daoud AS, Zaki M, Pugh RN, al-Mutairi G, Beseiso R, Nasrallah AY. Clinical presentation of enteric fever: its changing pattern in Kuwait. J TropMed Hyg. 1991;94(5):341-7.
19. Downey D, Anzimlt JC, Ellis-Pegler RB. Salmonella typhi infection in adults is not limited to travellers returning from the tropics. NZMed J. 1986;99(804):443-6.

20. Duff P, Engelsgjerd B. Typhoid fever on an obstetrics-gynecology service. AmJ ObstetGynecol. 1983;145(1):113-4.
21. Dutta TK, Beeresha, Ghotekar LH. Atypical manifestations of typhoid fever. JPostgradMed. 2001;47(4):248-51.
22. Goh KT, Teo SH, Tay L, Monteiro EH. Epidemiology and control of an outbreak of typhoid in a psychiatric institution. EpidemiolInfect. 1992;108(2):221-9.
23. Gupta S, Meena HS. Changing profile of enteric fever--in summer-91. J AssocPhysicians India. 1992;40(11):726-9.
24. Gupta SP, Gupta MS, Bhardwaj S, Chugh TD. Current clinical patterns of typhoid fever: a prospective study. J TropMed Hyg. 1985;88(6):377-81.
25. Gupta V, Kaur U, Singh G, Prakash C, Sharma M, Aggarwal KC. An outbreak of typhoid fever in Chandigarh, North India. TropGeogrMed. 1986;38(1):51-4.
26. Haq SA, Alam MN, Hossain SM, Ahmed T, Tahir M. Value of clinical features in the diagnosis of enteric fever. Bangladesh Med ResCouncBull. 1997;23(2):42-6.
27. Hoa NT, Diep TS, Wain J, Parry CM, Hien TT, Smith MD, et al. Community-acquired septicaemia in southern Viet Nam: the importance of multidrug-resistant Salmonella typhi. TransRSocTropMedHyg. 1998;92(5):503-8.
28. Hosoglu S, Geyik MF, Akalin S, Ayaz C, Kokoglu OF, Loeb M. A simple validated prediction rule to diagnose typhoid fever in Turkey. TransRSocTropMed Hyg. 2006;100(11):1068-74.
29. Hyams KC, Oldfield EC, Scott RM, Bourgeois AL, Gardiner H, Pazzaglia G, et al. Evaluation of febrile patients in Port Sudan, Sudan: isolation of dengue virus. AmJ TropMed Hyg. 1986;35(4):860-5.
30. Ispahani P, Slack RC. Enteric fever and other extraintestinal salmonellosis in University Hospital, Nottingham, UK, between 1980 and 1997. EurJ ClinMicrobiolInfectDis. 2000;19(9):679-87.
31. Kamat SA. Evaluation of therapeutic efficacy of trimethoprim-sulphamethoxazole and chloramphenicol in enteric fever. BrMedJ. 1970;3(5718):320-2.
32. Kamat SA, Herzog C. Typhoid: clinical picture and response to chloramphenicol. Prospective study in Bombay (1972). Infection. 1977;5(2):85-91.
33. Khan M, Coovadia Y, Connolly C, Sturm AW. Risk factors predicting complications in blood culture-proven typhoid fever in adults. ScandJ InfectDis. 2000;32(2):201-5.
34. Khan M, Coovadia YM, Connolly C, Sturm AW. Influence of sex on clinical features, laboratory findings, and complications of typhoid fever. AmJ TropMed Hyg. 1999;61(1):41-6.
35. Khan MA, Hayat Z, Sadick A. Ofloxacin in the treatment of typhoid fever resistant to chloramphenicol and amoxicillin. ClinTher. 1994;16(5):815-8.
36. Khosla SN. Typhoid hepatitis. PostgradMed J. 1990;66(781):923-5.
37. Khosla SN, Anand A, Singh U, Khosla A. Haematological profile in typhoid fever. TropDoct. 1995;25(4):156-8.
38. Khosla SN, Kalra IJ, Sharma A. Plasma cortisol levels in enteric fever. J AssocPhysicians India. 1989;37(10):643-5.
39. Khosla SN, Lochan R. Renal dysfunction in enteric fever. J AssocPhysicians India. 1991;39(5):382-4.
40. Khosla SN, Saman A, Khosla P, Sabharwal U, Khosla A. Drug resistant typhoid fever. TropDoct. 1998;28(4):235-7.

41. Khosla SN, Singh R, Singh GP, Trehan VK. The spectrum of hepatic injury in enteric fever. AmJ Gastroenterol. 1988;83(4):413-6.
42. Kuvandik C, Karaoglan I, Namiduru M, Baydar I. Predictive value of clinical and laboratory findings in the diagnosis of the enteric fever. New Microbiol. 2009 Jan;32(1):25-30.
43. Leung DT, Bogetz J, Itoh M, Ganapathi L, Pietroni MA, Ryan ET, et al. Factors associated with encephalopathy in patients with Salmonella enterica serotype Typhi bacteremia presenting to a diarrheal hospital in Dhaka, Bangladesh. Am J Trop Med Hyg. 2012 Apr;86(4):698-702
44. Liberti A, Loiacono L. Ciprofloxacin versus chloramphenicol in the treatment of salmonella infection. Int J AntimicrobAgents. 2000;16(3):347-8.
45. Lin FY, Becke JM, Groves C, Lim BP, Israel E, Becker EF, et al. Restaurant-associated outbreak of typhoid fever in Maryland: identification of carrier facilitated by measurement of serum Vi antibodies. J ClinMicrobiol. 1988;26(6):1194-7.
46. Mathura KC, Chaudhary D, Simkhada R, Pradhan M, Shrestha P, Gurubacharya DL. Study of clinical profile and antibiotic sensitivity pattern in culture positive typhoid fever cases. KathmanduUniv Med J (KUMJ). 2005;3(4):376-9.
47. Mathura KC, Gurubacharya DL, Shrestha A, Pant S, Basnet P, Karki DB. Clinical profile of typhoid patients. KathmanduUniv Med J (KUMJ). 2003;1(2):135-7.
48. Misra RC, Kulpati DD, Bala S, Prakash K, Gupta SK, Chuttani HK. Niridazole-in treatment of typhoid fever. Chemotherapy. 1971;16(5):326-31.
49. Morgenstern R, Hayes PC. The liver in typhoid fever: always affected, not just a complication. AmJ Gastroenterol. 1991;86(9):1235-9.
50. Mukhtar ED, Mekki MO. Trimethoprim-sulphamethoxazole in the treatment of enteric fever in the Sudan. TransRSocTropMed Hyg. 1981;75(6):771-3.
51. Nand N, Sharma M, Bhutani M, Singh GP, Sharma VK. Cardiac status in typhoid fever. Angiology. 1996;47(11):1095-100.
52. Nazar HS, Rabbani A, Riaz A, Anwar J. Presentation of typhoid fever patients in Hazara Division and response to different treatment regimens. J AyubMed CollAbbottabad. 2005;17(1):67-9.
53. Ndububa DA, Erhabor GE, Akinola DO. Typhoid and paratyphoid fever: a retrospective study. TropGastroenterol. 1992;13(2):56-63.
54. Neopane A, Singh SB, Bhatta R, Dhital B, Karki DB. Changing spectrum of antibiotic sensitivity in enteric fever. Kathmandu Univ Med J (KUMJ). 2008 Jan-Mar;6(1):12-5.
55. Okome-Nkoumou M, Ayo NE, Bekale J, Kombila M. [Typhoid and paratyphoid fever in adults in the Internal Medicine Department at Libreville (Gabon)]. Sante. 2000;10(3):205-9.
56. Olle-Goig JE, Ruiz L. Typhoid fever in rural Haiti. BullPan AmHealth Organ. 1993;27(4):382-8.
57. Panhotra BR, Saxena AK, Al-Ghamdi AM. Typhoid fever due to multiresistant Salmonella enterica serovar typhi having reduced susceptibility to ciprofloxacin and nalidixic acid resistance. SaudiMed J. 2004;25(10):1509-11.
58. Park SC, Lee CH, Kim SY, Park CH, Kim TW, Seok SE, et al. Clinical trial of cefotaxime in patients with typhoid fever. ClinTher. 1985;7(4):448-51.
59. Pohan HT. Clinical and laboratory manifestations of typhoid fever at Persahabatan Hospital, Jakarta. Acta Med Indones. 2004;36(2):78-83.

60. Rogerson SJ, Spooner VJ, Smith TA, Richens J. Hydrocortisone in chloramphenicol-treated severe typhoid fever in Papua New Guinea. TransRSocTropMed Hyg. 1991;85(1):113-6.
61. Sanford JP, Linh NN, Kutscher E, Arnold K, Gould K. Oxolinic acid in the treatment of typhoid fever due to chloramphenicol-resistant strains of Salmonella typhi. AntimicrobAgents Chemother. 1976;9(3):387-92.
62. Sardesai HV, Melinkere RD, Diwate AB. Trimethoprim-sulphamethoxazole in typhoid fever. TransRSocTropMed Hyg. 1971;65(2):189-94.
63. Shin BM, Paik IK, Cho HI. Bone marrow pathology of culture proven typhoid fever. J Korean Med Sci. 1994;9(1):57-63.
64. Shlim DR, Schwartz E, Eaton M. Clinical Importance of Salmonella Paratyphi A Infection to Enteric Fever in Nepal. JTravelMed. 1995;2(3):165-8.
65. Smith SI, Odunukwe NN, Niemogha MT, Ahmed AO, Efienemokwu CA, Otuonye MN, et al. Diagnostic methods for typhoid fever in Nigeria. BrJBiomedSci. 2004;61(4):179-81.
66. Spencer DC, Pienaar NL, Atkinson PM. Disturbances of blood coagulation associated with Salmonella typhi infections. J Infect. 1988;16(2):153-61.
67. Su CP, Chen YC, Chang SC. Changing characteristics of typhoid fever in Taiwan. J MicrobiolImmunolInfect. 2004;37(2):109-14.
68. Sulaiman K, Sarwari AR. Culture-confirmed typhoid fever and pregnancy. IntJInfectDis. 2007;11(4):337-41.
69. Torin DE, Betts SL, McClenahan JB, Phillips GM, Shikamura MT. A typhoid fever outbreak on a university campus. ArchInternMed. 1969;124(5):606-10.
70. Trevett AJ, Nwokolo N, Lightfoot D, Naraqi S, Kevau IH, Temu PI, et al. Ataxia in patients infected with Salmonella typhi phage type D2: clinical, biochemical and immunohistochemical studies. TransRSocTropMed Hyg. 1994;88(5):565-8.
71. Unal S, Hayran M, Tuncer S, Gur D, Uzun O, Akova M, et al. Treatment of enteric fever with pefloxacin for 7 days versus 5 days: a randomized clinical trial. AntimicrobAgents Chemother. 1996;40(12):2898-900.
72. Uwaydah AK, al SH, Matar I. Randomized prospective study comparing two dosage regimens of ciprofloxacin for the treatment of typhoid fever. J AntimicrobChemother. 1992;30(5):707-11.
73. Valenciano M, Baron S, Fisch A, Grimont F, Desenclos JC. Investigation of concurrent outbreaks of gastroenteritis and typhoid fever following a party on a floating restaurant, France, March 1998. AmJEpidemiol. 2000;152(10):934-9.
74. van den Bergh ET, Gasem MH, Keuter M, Dolmans MV. Outcome in three groups of patients with typhoid fever in Indonesia between 1948 and 1990. TropMed Int Health. 1999;4(3):211-5.
75. Walia M, Gaind R, Paul P, Mehta R, Aggarwal P, Kalaivani M. Age-related clinical and microbiological characteristics of enteric fever in India. TransRSocTropMed Hyg. 2006;100(10):942-8.
76. Walia M, Gaind R, Mehta R, Paul P, Aggarwal P, Kalaivani M. Current perspectives of enteric fever: a hospital-based study from India. AnnTropPaediatr. 2005;25(3):161-74.
77. Wang JL, Kao JH, Tseng SP, Teng LJ, Ho SW, Hsueh PR. Typhoid fever and typhoid hepatitis in Taiwan. EpidemiolInfect. 2005;133(6):1073-9.
78. Wallace MR, Yousif AA, Mahroos GA, Mapes T, Threlfall EJ, Rowe B, et al. Ciprofloxacin versus ceftriaxone in the treatment of multiresistant typhoid fever. EurJ ClinMicrobiolInfectDis. 1993;12(12):907-10.

79. Yap PK, Chua CT. The haemogram in the diagnosis of acute typhoid fever--with special reference to thrombocytopenia. Singapore Med J. 1983;24(3):161-2.

- **<u>Table S4: Spectrum of enteric fever in children by economic regions and geographical regions</u>**

	**High Income countries** 17 studies			**Europe& Central Asia** 4 studies			**Sub-Saharan Africa** 12 studies			**Middle East & North Africa** 3 studies			**South Asia** 37 studies			**East Asia & Pacific** 10 studies		
**Demographics/History**	**N**	**N**	**%**	**n**	**N**	**%**	**n**	**N**	**%**	**N**	**N**	**%**	**n**	**N**	**%**	**n**	**N**	**%**
Males	262	472	**56**	77	131	**59**	574	1077	**53**	162	281	**58**	2626	4512	**58**	371	661	**56**
Duration of illness 1-2weeks	63	161	**39**				109	420	**26**	40	150	**27**	556	1712	**32**	63	158	**40**
Duration of illness > 2 weeks	74	161	**46**				35	320	**11**	18	150	**12**	432	1863	**23**	5	158	**3**
Duration of illness > 1 week	63	134	**47**				131	424	**31**				672	1380	**49**	122	325	**38**
Chloramphenicol resistance	10	257	**4**	2	83	**2**	0	120	**0**				712	1075	**66**	66	426	**15**
Ampicillin/Amoxycillin resistance	19	245	**8**	3	83	**4**	6	120	**5**				506	781	**65**	41	329	**12**
Co-trimoxazole resistance	17	245	**7**	2	83	**2**	10	181	**6**				502	708	**71**	29	337	**9**
**Laboratory features**																		
Severe anaemia (<7.5 g/dl)							52	152	**34**	3	62	**5**	3	50	**6**	7	163	**4**
Neutrophilia(>7.5 x 10^3/µL)				21	24	**88**							72	131	**55**	116	177	**66**
Thrombocytopenia (<150 x 10^3/µL)	39	264	**15**	0	15	**0**	92	266	**35**				53	1208	**4**	47	173	**27**
Severe thrombocytopenia (<50 x 10^3/µL)							4	91	**4**	1	71	**1**				5	17	**29**
Hemolysis or hemolytic anaemia	11	127	**9**	1	27	**4**	2	144	**1**							7	279	**3**
**Other signs and symptoms**																		
Chills or rigors	40	106	**38**							4	71	**6**	154	503	**31**	83	638	**13**
Anorexia/refusal to feed	140	279	**50**	37	72	**51**	73	224	**33**				770	3184	**24**	246	502	**49**
Weakness or malaise	58	61	**95**	23	51	**45**	6	217	**3**				48	161	**30**	9	30	**30**
Continuous fever							3	54	**6**	31	150	**21**	262	351	**75**	32	137	**23**
Intermittent fever	96	127	**76**				11	35	**31**	5	150	**3**	171	354	**48**	52	137	**38**
Remittent fever	28	111	**25**				64	231	**28**	114	150	**76**	29	245	**12**	51	137	**37**
Stepladder fever													0	93	**0**	0	54	**0**
Chelosis	2	24	**8**										33	447	**7**	4	137	**3**
URTI	31	111	**28**				11	47	**23**	37	150	**25**	436	2765	**16**	19	314	**6**
**Other complications**																		
Myocarditis							1	107	**1**				24	908	**3**	12	397	**3**
Meningitis or meningismus	18	326	**6**				170	957	**18**	12	221	**5**	54	1231	**4**	9	488	**2**
Hepatitis or raised LFTs	23	56	**41**	13	43	**30**	13	296	**4**				199	2427	**8**	70	173	**40**
Jaundice	4	181	**2**	3	51	**6**	33	569	**6**				109	3485	**3**	12	538	**2**
DIC													91	336	**27**	1	54	**2**
Acute acalculouscholecystitis	1	28	**4**										39	970	**4**	4	156	**3**
Edema/pleural effusion/ascites	8	83	**10**				4	117	**3**				14	674	**2**	6	193	**3**
Convulsions	2	58	**3**	3	27	**11**	7	324	**2**				22	590	**4**	4	30	**13**
Focal neurological involvement	4	153	**3**	3	51	**6**	11	385	**3**	5	71	**7**	65	2249	**3**	17	458	**4**
Epistaxis/bleeding(unspecified)	24	240	**10**				3	117	**3**				33	772	**4**	16	242	**7**
Any abscess	1	50	**2**				3	164	**2**				2	488	**0**			

Osteomyelitis or septic arthritis	1	206	**0**				10	184	**5**				2	625	**0**	2	102	**2**
Renal involvement	4	16	**25**				59	974	**6**	20	71	**28**	8	823	**1**	46	116	**40**
Arthritis or arthralgia or myalgia	24	152	**16**	40	96	**42**	17	271	**6**	2	71	**3**	60	1182	**5**	16	384	**4**

Table S5: Spectrum of enteric fever in children in Latin America & Caribbean

	**Latin America & Caribbean** 2 studies		
	**n**	**N**	**%**
Males	8	13	**62**
Blood/bone marrow Isolates	13	13	**100**
Duration of illness≤ 1week	4	4	**100**
Fever	4	4	**100**
High grade fever	0	4	**0**
Anorexia/refusal to feed	2	4	**50**
Cough	4	4	**100**
Nausea or vomiting	2	4	**50**
Diarrhea	0	4	**0**
Constipation	0	4	**0**
Hepatomegaly	0	4	**0**
Splenomegaly	0	4	**0**
Anaemia (Hb < 12 g/dl)	9	9	**100**
Neutrophilia(>7.5 x 10^3/µL)	9	9	**100**
Meningitis or meningismus	1	9	**11**
Pneumonia or chest signs	1	9	**11**
Hepatitis or raised LFTs	2	9	**22**
Epistaxis/bleeding	4	9	**44**
Death	2	13	**15**

- Table S6: Associations of clinical features in enteric fever by geographical regions (Africa* versus South Asia)

Clinical Features	Odds Ratio	95% Confidence Interval
Multi-drug resistant isolate	0.6	0.5 - 0.8
Toxicity	2.2	1.8 - 2.7
Diarrhea	2.3	2.0 - 2.6
Constipation	3.3	2.4 - 4.5
Relative bradycardia	15.3	9.6 - 24.3
Leucopenia (< 5 x 10^3/µL)	2.8	2.1 - 3.6
Leucocytosis (> 15)	0.6	0.4 - 0.9
Thrombocytopenia	11.5	7.9 - 16.7
Abdominal pain/tenderness	2.4	2.1 - 2.8
Abdominal distention or ileus	7.4	6.2 - 8.9
Altered mental status	1.5	1.2 - 1.8
Meningitis or meningismus	4.1	3.0 - 5.5
Pneumonia	3.1	2.6- 3.8
GI bleeding	0.5	0.3 - 0.8
Death	3.6	2.7 - 4.9
Severe anaemia (<7.5)	5.4	1.6 - 18.1
URTI	1.7	1.2 - 2.3

Duration of illness > 2 weeks	0.4	0.3 - 0.6
Duration of illness > 1 week	0.8	0.6 - 1.0
Chills or rigors	0.2	0.1 - 0.6
Anorexia/refusal to feed	1.5	1.1 - 2.0
Weakness or malaise	0.07	0.03 - 0.2
Continuous fever	0.07	0.04 - 0.1
Remittent fever	6.5	4.2- 10.1
Intermittent fever	0.1	0.06 - 0.2
Hepatitis or raised LFTs	0.5	0.3 - 0.9
Jaundice	1.9	1.3- 2.9
Osteomyelitis or septic arthritis	17.9	3.9 - 82.5
Renal involvement	7.8	4.0 - 15.1

* Sub-Saharan Africa (12 studies) and Middle East & North Africa (3 studies)

References to Table 2, Table S4, Table S5 and Table S6: Spectrum of enteric fever in children by economic regions

High Income Countries

1. Chiu CH, Lin TY. Typhoid fever in children. Lancet. 1999;354(9194):2001-2.
2. Chiu CH, Tsai JR, Ou JT, Lin TY. Typhoid fever in children: a fourteen-year experience. Acta PaediatrTaiwan. 2000;41(1):28-32.
3. Chow CB, Wang PS, Leung NK. Typhoid fever in Hong Kong children. AustPaediatrJ. 1989;25(3):147-50.
4. Collins RN, Marine WM, Nahmias AJ. The 1964 epidemic of typhoid fever in Atlanta. Clinical and epidemiologic observations. JAMA. 1966;197(3):179-84.
5. Colon AR, Gross DR, Tamer MA. Typhoid fever in children. Pediatrics. 1975;56(4):606-9.
6. Colon AR. Gastrointestinal phenomena in childhood typhoid fever. SouthMed J. 1976;69(7):914.
7. Daoud AS, Zaki M, Pugh RN, al-Mutairi G, Beseiso R, Nasrallah AY. Clinical presentation of enteric fever: its changing pattern in Kuwait. J TropMed Hyg. 1991;94(5):341-7
8. Del Rosario AJ, Haynes RE, Cramblett HG. Typhoid fever in children. OhioState Med J. 1971;67(6):516-20.
9. Galloway H, Clark NS, Blackhall M. Paediatric aspects of the Aberdeen typhoid outbreak. ArchDisChild. 1966;41(215):63-8.
10. Hardy G, Padfield CJ, Chadwick P, Partington MW. Typhoid outbreak in Kingston, Ont: experience with high-dose oral ampicillin. CanMed AssocJ. 1977;116(7):761-4, 7.
11. Ispahani P, Slack RC. Enteric fever and other extraintestinal salmonellosis in University Hospital, Nottingham, UK, between 1980 and 1997. EurJ ClinMicrobiolInfectDis. 2000;19(9):679-87.
12. Mallouh AA, Sa'di AR. White blood cells and bone marrow in typhoid fever. Pediatr InfectDisJ. 1987;6(6):527-9.

13. Misra S, Diaz PS, Rowley AH. Characteristics of typhoid fever in children and adolescents in a major metropolitan area in the United States. ClinInfectDis. 1997;24(5):998-1000.
14. Oh HM, Masayu Z, Chew SK. Typhoid fever in hospitalized children in Singapore. J Infect. 1997;34(3):237-42.
15. Papaevangelou V, Syriopoulou V, Charissiadou A, Pangalis A, Mostrou G, Theodoridou M. Salmonella bacteraemia in a tertiary children's hospital. ScandJ InfectDis. 2004;36(8):547-51.
16. Stormon MO, McIntyre PB, Morris J, Fasher B. Typhoid fever in children: diagnostic and therapeutic difficulties. Pediatr InfectDisJ. 1997;16(7):713-4.
17. Su CP, Chen YC, Chang SC. Changing characteristics of typhoid fever in Taiwan. J MicrobiolImmunolInfect. 2004;37(2):109-14.
18. Tolaymat A, Fakhreddine F, David CB, Whitworth JM. Typhoid fever in children: a forgotten disease? SouthMed J. 1979;72(2):136-8.
19. Wang JL, Kao JH, Tseng SP, Teng LJ, Ho SW, Hsueh PR. Typhoid fever and typhoid hepatitis in Taiwan. EpidemiolInfect. 2005;133(6):1073-9.

Sub Saharan Africa:

1. Ali Omer MI. Trimethoprim-sulphamethoxazole in the treatment of enteric fever. JTropMedHyg. 1975;78(7):162-6.
2. Duggan MB, Beyer L. Enteric fever in young Yoruba children. ArchDisChild. 1975;50(1):67-71
3. Ellis ME, Moosa A, Hillier V. A review of typhoid fever in South African black children. PostgradMed J. 1990;66(782):1032-6
4. Green SD, Cheesbrough JS. Salmonella bacteraemia among young children at a rural hospital in western Zaire. AnnTropPaediatr. 1993;13(1):45-53.
5. Johnson AO, Aderele WI. Enteric fever in childhood. J TropMed Hyg. 1981;84(1):29-35.
6. Kagwa-Nyanzi JA. Typhoid fever in African children. J TropPediatr EnvironChild Health. 1971;17(3):105-9.
7. Khan M, Coovadia YM, Connolly C, Sturm AW. Influence of sex on clinical features, laboratory findings, and complications of typhoid fever. AmJ TropMed Hyg. 1999;61(1):41-6.
8. Laditan AA, Alausa KO. Problems in the clinical diagnosis of typhoid fever in children in the tropics. AnnTropPaediatr. 1981;1(3):191-5.
9. Lepage P, Bogaerts J, Van GC, Ntahorutaba M, Nsengumuremyi F, Hitimana DG, et al. Community- acquired bacteraemia in African children. Lancet. 1987;1(8548):1458-61
10. Mtove G, Amos B, von Seidlein L, Hendriksen I, Mwambuli A, Kimera J, et al. Invasive salmonellosis among children admitted to a rural Tanzanian hospital and a comparison with previous studies. PLoS One. 2010;5(2):e9244.
11. Mulligan TO. Typhoid fever in young children. BrMed J. 1971;4(5788):665-7
12. Nesbitt A, Mirza NB. Salmonella septicaemias in Kenyan children. J TropPediatr. 1989;35(1):35-9
13. Scragg JN, Rubidge CJ. Trimethoprim and sulphamethoxazole in typhoid fever in children. BrMed J. 1971;3(5777):738-41.
14. Topley JM. Mild typhoid fever. ArchDisChild. 1986;61(2):164-7.

East Asia and Pacific:

1. Brian MJ. Typhoid in children in Goroka. PNGMed J. 1990;33(1):68-9.
2. Choo KE, Razif A, Ariffin WA, Sepiah M, Gururaj A. Typhoid fever in hospitalized children in Kelantan, Malaysia. AnnTropPaediatr. 1988;8(4):207-12.
3. Emary K, Moore CE, Chanpheaktra N, An KP, Chheng K, Sona S, et al. Enteric fever in Cambodian children is dominated by multidrug-resistant H58 Salmonella enterica serovar Typhi with intermediate susceptibility to ciprofloxacin. Trans R Soc Trop Med Hyg. 2012 Dec;106(12):718-24.
4. Hoa NT, Diep TS, Wain J, Parry CM, Hien TT, Smith MD, et al. Community-acquired septicaemia in southern Viet Nam: the importance of multidrug-resistant Salmonella typhi.
5. Malik AS, Malik RH. Typhoid fever in Malaysian children. Med J Malaysia. 2001;56(4):478-90.
6. Malik AS. Complications of bacteriologically confirmed typhoid fever in children. J TropPediatr. 2002;48(2):102-8
7. Soelistyowati S, Soenarto Y, Soesilo H, Widiarto, Widiatmodjo, Ismangoen. Typhoid fever in children. Paediatr Indones. 1982;22(7-8):138-46.
8. Shwe TN, Nyein MM, Yi W, Mon A. Blood culture isolates from children admitted to Medical Unit III, Yangon Children's Hospital, 1998. Southeast Asian J TropMed
9. Thisyakorn U, Mansuwan P, Taylor DN. Typhoid and paratyphoid fever in 192 hospitalized children in Thailand. AmJ DisChild. 1987;141(8):862-5
10. Thisyakorn U, Mansuwan P. Comparative efficacy of mecillinam, mecillinam/amoxicillin and trimethoprim-sulfamethoxazole for treatment of typhoid fever in children. PediatrInfectDisJ.
11. Wongsawat J, Pancharoen C, Thisyakorn U. Typhoid fever in children: experience in King Chulalongkorn Memorial Hospital. J Med AssocThai. 2002;85
12. Yap YF, Puthucheary SD. Typhoid fever in children--a retrospective study of 54 cases from Malaysia. Singapore Med J. 1998;39(6):260-2.

Europe and Central Asia:

1. Caglar MK, Ozsoylu S, Kanra G. Relative granulocytosis in childhood typhoid fever. J Pediatr. 1983;102(4):603-4
2. Ciftci E, Guriz H, Derya AA, Ince E, Erdem B, Dogru U. Salmonella bacteraemia in Turkish children: 37 cases seen in a university hospital between 1993 and 2002.
3. Kanra G, Yurdakok M, Secmeer G, Arvas A, Dereli O, Sarikayalar F, et al. Clinical findings of pediatric patients during an outbreak of typhoid fever in Ankara. TurkJ
4. Secmeer G, Kanra G, Cemeroglu AP, Ozen H, Ceyhan M, Ecevit Z. Salmonella typhi infections. A 10-year retrospective study. TurkJ Pediatr. 1995;37(4):339-41.
5. Tatli MM, Aktas G, Kosecik M, Yilmaz A. Treatment of typhoid fever in children with a flexible-duration of ceftriaxone, compared with 14-day treatment with chloramphenicol.

Latin America:

1. Ferreccio C, Levine MM, Manterola A, Rodriguez G, Rivara I, Prenzel I, et al. Benign bacteremia caused by Salmonella typhi and paratyphi in children younger than 2
2. Palacios Malmaceda PG, Vela Acosta JJ, Gutierez AW. [Typhoid fever in children under 2 years of age]. BolMedHospInfant Mex. 1981;38(3):473-83

Middle East and North Africa:

1. Girgis NI, Tribble DR, Sultan Y, Farid Z. Short course chemotherapy with cefixime in children with multidrug-resistant Salmonella typhi Septicaemia. J TropPediatr. 1995;41(6):364-5
2. Khuri-Bulos N. Enteric fevers in children. The importance of age in the varying clinical picture. ClinPediatr (Phila). 1981;20(7):448-52.
3. Moussa K. Enterica of childhood. J EgyptPublic Health Assoc. 1970;45(1):48-55.

South Asia:
1. Agarwal KS, Singh SK, Kumar N, Srivastav R, Rajkumar A. A study of current trends in enteric fever. J CommunDis. 1998;30(3):171-4.
2. Ahmad KA, Khan LH, Roshan B, Bhutta ZA. Factors associated with typhoid relapse in the era of multiple drug resistant strains. J Infect Dev Ctries. 2011 Oct;5(10):727-31.
3. Arora RK, Gupta A, Joshi NM, Kataria VK, Lall P, Anand AC. Multidrug resistant typhoid fever: study of an outbreak in Calcutta. Indian Pediatr. 1992;29(1):61-6.
4. Bhutta ZA. Impact of age and drug resistance on mortality in typhoid fever. ArchDisChild. 1996;75(3):214-7.
5. Bhutta ZA. Fulminant hepatic failure with typhoid fever in childhood. J Pak Med Assoc. 1991;41(6):123-6.
6. Bhutta ZA, Mansoorali N, Hussain R. Plasma cytokines in paediatric typhoidal salmonellosis: correlation with clinical course and outcome. J Infect. 1997;35(3):253-6.
7. Bhutta ZA, Naqvi SH. Hepatic dysfunction in paediatric typhoidal salmonellosis. AnnTropPaediatr. 1991;11(3):203-5.
8. Bhutta ZA, Naqvi SH, Durrani S, Suria A. Chloramphenicol therapy of typhoid fever and its relationship to hepatic dysfunction. JTropPediatr. 1991;37(6):320-2.
9. Bhutta ZA, Naqvi SH, Razzaq RA, Farooqui BJ. Multidrug-resistant typhoid in children: presentation and clinical features. RevInfectDis. 1991;13(5):832-6.
10. Bhutta ZA, Niazi SK, Suria A. Chloramphenicol clearance in typhoid fever: implications for therapy. Indian J Pediatr. 1992;59(2):213-9
11. Bhutta ZA. Enteric Fever: Risk Factors for Treatment Failure in the Era of Multidrug Resistance. UNPUBLISHED.
12. Biswal N. Neurological manifestations of typhoid fever in children. J TropPediatr. 1994;40(3):190.
13. Biswal N, Mathai B, Bhatia BD, Srinivasan S, Nalini P. Enteric fever: a changing perspective. Indian Pediatr. 1994;31(7):813-9.
14. Buch NA, Hassan MU, Kakroo DK. Enteric fever--a changing sensitivity pattern, clinical profile and outcome. Indian Pediatr. 1994;31(8):981-5
15. Chandra R, Srinivasan S, Nalini P, Rao RS. Multidrug resistant enteric fever. J TropMed Hyg. 1992;95(4):284-7.
16. Chawla V, Bhujwala RA, Chandra RK, Ghai OP. Typhoid fever in children. Clinical and bacteriological studies. Indian J Pediatr. 1970;37(269):239-44.
17. Dheer G, Kundra S, Singh T. Clinical and laboratory profile of enteric fever in children in northern India. Trop Doct. 2012 Jul;42(3):154-6.
18. Dutta P, Lahiri M, Bhattacharya SK, Saha MR, Mitra U, Rasaily R, et al. Hepatitis-like presentation in typhoid fever. TransRSocTropMed Hyg. 1992;86(1):92.

19. Dutta P, Rasaily R, Saha MR, Mitra U, Manna B, Chakraborty S, et al. Randomized clinical trial of furazolidone for typhoid fever in children. ScandJGastroenterol. 1993;28(2):168-72.
20. Garg K, Mangal N, Mathur HC. Clinical profile of multi drug resistant typhoid fever in Jaipur City. Indian Pediatr. 1994;31(2):191-3.
21. Garg RA, Krashak R. Typhoid fever before two years of age. Indian Pediatr. 1993;30(6):805-8
22. Gulati S, Marwaha RK, Singhi S, Ayyagari A, Kumar L. Third generation cephalosporins in multi-drug resistant typhoid fever. Indian Pediatr. 1992;29(4):513-6.
23. Hassan M. Multiply-resistant Salmonella typhi in children. Drugs. 1995;49 Suppl 2:457-9.
24. Jagadish K, Patwari AK, Sarin SK, Prakash C, Srivastava DK, Anand VK. Hepatic manifestations in typhoid fever. Indian Pediatr. 1994;31(7):807-11.
25. Jain S, Chitnis DS, Sham A, Rathi S, Inamdar S, Rindani GJ. Outbreak of chloramphenicol resistant typhoid fever. Indian Pediatr. 1987;24(3):193-7
26. Kabra SK, Madhulika, Talati A, Soni N, Patel S, Modi RR. Multidrug-resistant typhoid fever. TropDoct. 2000;30(4):195-7.
27. Kapoor JP, Mohan M, Talwar V, Daral TS, Bhargava SK. Typhoid fever in young children. Indian Pediatr. 1985;22(11):811-3.
28. Khan MI, Soofi SB, Ochiai RL, Khan MJ, Sahito SM, Habib MA, et al. Epidemiology, clinical presentation, and patterns of drug resistance of Salmonella Typhi in Karachi, Pakistan. J Infect Dev Ctries. 2012 Oct;6(10):704-14.
29. Koul PB, Murali MV, Sharma PP, Ghai OP, Ramchandran VG, Talwar V. Multi drug resistant Salmonella typhi infection: clinical profile and therapy. Indian Pediatr. 1991;28(4):357-61.
30. Kulkarni ML, Rego SJ. Acute acalculous cholecystitis in typhoid fever. Indian Pediatr. 1995;32(7):829-30
31. Kumar R, Gupta N. Multidrug-resistant typhoid fever. Indian J Pediatr. 2007;74(1):39-42.
32. Leung DT, Bogetz J, Itoh M, Ganapathi L, Pietroni MA, Ryan ET, et al. Factors associated with encephalopathy in patients with Salmonella enterica serotype Typhi bacteremia presenting to a diarrheal hospital in Dhaka, Bangladesh. Am J Trop Med Hyg. 2012 Apr;86(4):698-702
33. Libov A. Enteric fever in children. Indian JPediatr. 1960;27:125-8.Mishra S, Patwari AK, Anand VK, Pillai PK, Aneja S, Chandra J, et al. A clinical profile of multidrug resistant typhoid fever. Indian Pediatr. 1991;28(10):1171-4.
34. Memon IA, Billoo AG, Memon HI. Cefixime: an oral option for the treatment of multidrug-resistant enteric fever in children. SouthMed J. 1997;90(12):1204-7.
35. Mishra S, Patwari AK, Anand VK, Pillai PK, Aneja S, Chandra J, et al. A clinical profile of multidrug resistant typhoid fever. Indian Pediatr. 1991;28(10):1171-4.
36. Naeemullah S. A 3 year study of children with enteric fever. Pakistan Pediatric Journal. 1994;18(4):165-9.
37. Owais A, Sultana S, Zaman U, Rizvi A, Zaidi AK. Incidence of typhoid bacteremia in infants and young children in southern coastal Pakistan. Pediatr Infect Dis J. 2010 Nov;29(11):1035-9.
38. Pradhan R, Shrestha U, Gautam SC, Thorson S, Shrestha K, Yadav BK, et al. Bloodstream infection among children presenting to a general hospital outpatient clinic in urban Nepal. PLoS One. 2012;7(10):e47531

39. Rajajee S, Anandi TB, Subha S, Vatsala BR. Patterns of resistant Salmonella typhi infection in infants. JTropPediatr. 1995;41(1):52-4.
40. Raman TS, Krishnamurthy L, Menon PK, Singh D, Jayaprakash DG. Clinical profile and therapy in enteric fever. Indian Pediatr. 1994;31(2):196-9.
41. Ramanan A, Pandit N, Yeshwanth M. Unusual complications in a multidrug resistant Salmonella typhi outbreak. Indian Pediatr. 1992;29(1):118-20.
42. Rasaily R, Dutta P, Saha MR, Mitra U, Lahiri M, Pal SC. Multi-drug resistant typhoid fever in hospitalised children. Clinical, bacteriological and epidemiological profiles.
43. Rathore MH, Bux D, Hasan M. Multidrug-resistant Salmonella typhi in Pakistani children: clinical features and treatment. SouthMed J. 1996;89(2):235-7.
44. Sethuraman S, Mahamood M, Kareem S. Furazolidone in multi-resistant childhood typhoid fever. AnnTropPaediatr. 1994;14(4):321-4.
45. Sharma A, Gathwala G. Clinical profile and outcome in enteric fever. Indian Pediatr. 1993;30(1):47-50.
46. Shetty AK, Mital SR, Bahrainwala AH, Khubchandani RP, Kumta NB. Typhoid hepatitis in children. J TropPediatr. 1999;45(5):287-90.
47. Siddiqui FJ, Rabbani F, Hasan R, Nizami SQ, Bhutta ZA. Typhoid fever in children: some epidemiological considerations from Karachi, Pakistan. Int J
48. Singh H, Raizada N. Chloramphenicol resistant typhoid fever. Indian Pediatr. 1991;28(4):433.
49. Sinha A, Sazawal S, Kumar R, Sood S, Reddaiah VP, Singh B, et al. Typhoid fever in children aged less than 5 years. Lancet. 1999;354(9180):734-7.
50. Smith M. Typhoid admissions in childhood. TropDoct. 1986;16(4):150-2
51. Takkar VP, Kumar R, Takkar R, Khurana S. Resurgence of chloramphenicol sensitive Salmonella typhi. Indian Pediatr. 1995;32(5):586-7.
52. Thambidorai CR, Shyamala J, Sarala R, Vatsala RB, Tamizhisai S. Acute acalculous cholecystitis associated with enteric fever in children. Pediatr Infect Dis J. 1995 Sep;14(9):812-3.
53. Verma PS, Mullick P, Ghosh S. Neurological manifestations of enteric fever: clinical profile and correlation with ultimate outcome. Indian Pediatr. 1972;9(11):681-5.
54. Walia M, Gaind R, Mehta R, Paul P, Aggarwal P, Kalaivani M. Current perspectives of enteric fever: a hospital-based study from India. AnnTropPaediatr. 2005;25(3):161-74.
55. Mishra S, Patwari AK, Anand VK, Pillai PK, Aneja S, Chandra J, et al. A clinical profile of multidrug resistant typhoid fever. Indian Pediatr. 1991;28(10):1171-4.

- Table S7: Enteric fever in children in hospital-based versus community-based setting

	**Hospital-based** 80 studies			**Community-based or health center (out-patients)** 5 studies		
**Demographics/History**	**N**	**N**	**%**	**n**	**N**	**%**
Males	4075	7142	**57**	2	4	**50**
Duration of illness within 1-2weeks	831	2601	**32**			

Duration of illness > 2 weeks	564	2652	**21**			
Duration of illness > 1 week	988	2263	**44**			
Chloramphenicol resistance	780	1895	**41**	10	28	**36**
Ampicillin/Amoxycillin resistance	567	1520	**37**			
Co-trimoxazole resistance	547	1516	**36**			
**Laboratory features**						
Severe anaemia (<7.5 g/dl)	53	393	**13**			
Neutrophilia(>7.5 x 10^3/µL)	218	341	**64**			
Thrombocytopenia (<150 x 10^3/µL)	231	1926	**12**			
Severe thrombocytopenia (<50 x 10^3/µL)	10	179	**6**			
Hemolysis or hemolytic anaemia	21	577	**4**			
**Other signs and symptoms**						
Chills or rigors	281	1318	**21**			
Anorexia/refusal to feed	1264	4212	**30**	4	53	**8**
Weakness/dizziness or malaise	142	471	**30**	2	49	**4**
Continuous fever	328	692	**47**			
Intermittent fever	335	803	**42**			
Remittent fever	286	874	**33**			
Stepladder fever	0	147	**0**			
Chelosis	39	608	**6**			
URTI	532	3371	**16**	2	16	**13**
**Other complications**						
Myocarditis	37	1412	**3**			
Meningitis or meningismus	260	3160	**8**			
Hepatitis or raised LFTs	320	3004	**11**			
Jaundice	156	4790	**3**			
DIC	92	390	**24**			
Acute acalculouscholecystitis	44	1154	**4**			
Edema or pleural effusion or ascites	32	1067	**3**			
Convulsions	103	3329	**3**			
Focal neurological involvement	38	1029	**4**			
Epistaxis/bleeding (unspecified)	79	1342	**6**			
Any abscess	6	702	**1**			
Osteomyelitis or septic arthritis	11	1083	**1**			
Renal involvement	137	2000	**7**			
Arthritis or arthralgia or myalgia	159	2156	**7**			

- Table S8: Associations of clinical features in enteric fever in children by setting (hospital-based versus community/health-center)

Clinical Features	Odds Ratio	95% Confidence Interval
Pre-treatment antibiotics received	2.8	2.0 - 4.0
Multidrug resistant isolates	1.7	1.3 - 2.1
High - grade fever	4.7	3.5 - 6.4
Headache	0.3	0.2 - 0.4
Toxicity	0.3	0.1 - 0.6
Nausea/vomiting	2.0	1.6 - 2.6
Cough	0.7	0.5 - 0.9
Diarrhea	5.2	3.8 - 7.2

Constipation	4.2	1.9 - 9.6
Hepatomegaly	7.1	4.2 - 12.0
Splenomegaly	2.7	1.7 - 4.1
Abdominal pain/tenderness	0.5	0.4 - 0.6
Abdominal distention or ileus	7.4	2.7 - 20.0
GI bleeding	9.0	1.2 - 64.3
Duration of illness ≤ 1week	1.8	1.3 - 2.4
Fluoroquinolone resistance	0.03	0.01 - 0.08

References for Table 3 , Table S7, and Table S8: Typhoid fever in children in hospital-based vs. community/health-center

Community Based:
1. Ferreccio C, Levine MM, Manterola A, Rodriguez G, Rivara I, Prenzel I, et al. Benign bacteremia caused by Salmonella typhi and paratyphi in children younger than 2 years. J Pediatr. 1984;104(6):899-901.
2. Khan MI, Soofi SB, Ochiai RL, Khan MJ, Sahito SM, Habib MA, et al. Epidemiology, clinical presentation, and patterns of drug resistance of Salmonella Typhi in Karachi, Pakistan. J Infect Dev Ctries. 2012 Oct;6(10):704-14.
3. Owais A, Sultana S, Zaman U, Rizvi A, Zaidi AK. Incidence of typhoid bacteremia in infants and young children in southern coastal Pakistan. Pediatr Infect Dis J. 2010
4. Pradhan R, Shrestha U, Gautam SC, Thorson S, Shrestha K, Yadav BK, et al. Bloodstream infection among children presenting to a general hospital outpatient clinic in urban Nepal. PLoS One. 2012;7(10):e47531
5. Sinha A, Sazawal S, Kumar R, Sood S, Reddaiah VP, Singh B, et al. Typhoid fever in children aged less than 5 years. Lancet. 1999;354(9180):734-7.
6. Siddiqui FJ, Rabbani F, Hasan R, Nizami SQ, Bhutta ZA. Typhoid fever in children: some epidemiological considerations from Karachi, Pakistan. Int J InfectDis. 2006;10(3):215-22.

Hospital Based:
1. Ali Omer MI. Trimethoprim-sulphamethoxazole in the treatment of enteric fever. JTropMedHyg. 1975;78(7):162-6.
2. Ahmad KA, Khan LH, Roshan B, Bhutta ZA. Factors associated with typhoid relapse in the era of multiple drug resistant strains. J Infect Dev Ctries. 2011 Oct;5(10):727-31.
3. Arora RK, Gupta A, Joshi NM, Kataria VK, Lall P, Anand AC. Multidrug resistant typhoid fever: study of an outbreak in Calcutta. Indian Pediatr. 1992;29(1):61-6.
4. Bhutta ZA. Impact of age and drug resistance on mortality in typhoid fever. ArchDisChild. 1996;75(3):214-7.
5. Bhutta ZA. Fulminant hepatic failure with typhoid fever in childhood. J Pak Med Assoc. 1991;41(6):123-6.
6. Bhutta ZA, Mansoorali N, Hussain R. Plasma cytokines in paediatric typhoidal salmonellosis: correlation with clinical course and outcome. J Infect. 1997;35(3):253-6.

7. Bhutta ZA, Naqvi SH. Hepatic dysfunction in paediatric typhoidal salmonellosis. AnnTropPaediatr. 1991;11(3):203-5.
8. Bhutta ZA, Naqvi SH, Durrani S, Suria A. Chloramphenicol therapy of typhoid fever and its relationship to hepatic dysfunction. JTropPediatr. 1991;37(6):320-2.
9. Bhutta ZA, Naqvi SH, Razzaq RA, Farooqui BJ. Multidrug-resistant typhoid in children: presentation and clinical features. RevInfectDis. 1991;13(5):832-6.
10. Bhutta ZA, Niazi SK, Suria A. Chloramphenicol clearance in typhoid fever: implications for therapy. Indian J Pediatr. 1992;59(2):213-9
11. Bhutta ZA. Enteric Fever: Risk Factors for Treatment Failure in the Era of Multidrug Resistance. UNPUBLISHED.
12. Biswal N. Neurological manifestations of typhoid fever in children. J TropPediatr. 1994;40(3):190.
13. Biswal N, Mathai B, Bhatia BD, Srinivasan S, Nalini P. Enteric fever: a changing perspective. Indian Pediatr. 1994;31(7):813-9.
14. Brian MJ. Typhoid in children in Goroka. PNGMed J. 1990;33(1):68-9.
15. Buch NA, Hassan MU, Kakroo DK. Enteric fever--a changing sensitivity pattern, clinical profile and outcome. Indian Pediatr. 1994;31(8):981-5
16. Caglar MK, Ozsoylu S, Kanra G. Relative granulocytosis in childhood typhoid fever. J Pediatr. 1983;102(4):603-4
17. Chandra R, Srinivasan S, Nalini P, Rao RS. Multidrug resistant enteric fever. J TropMed Hyg. 1992;95(4):284-7.
18. Chawla V, Bhujwala RA, Chandra RK, Ghai OP. Typhoid fever in children. Clinical and bacteriological studies. Indian J Pediatr. 1970;37(269):239-44.
19. Chiu CH, Lin TY. Typhoid fever in children. Lancet. 1999;354(9194):2001-2.
20. Chiu CH, Tsai JR, Ou JT, Lin TY. Typhoid fever in children: a fourteen-year experience. Acta PaediatrTaiwan. 2000;41(1):28-32.
21. Choo KE, Razif A, Ariffin WA, Sepiah M, Gururaj A. Typhoid fever in hospitalized children in Kelantan, Malaysia. AnnTropPaediatr. 1988;8(4):207-12.
22. Chow CB, Wang PS, Leung NK. Typhoid fever in Hong Kong children. AustPaediatrJ. 1989;25(3):147-50.
23. Ciftci E, Guriz H, Derya AA, Ince E, Erdem B, Dogru U. Salmonella bacteraemia in Turkish children: 37 cases seen in a university hospital between 1993 and 2002. AnnTropPaediatr. 2004;24(1):75-80..
24. Collins RN, Marine WM, Nahmias AJ. The 1964 epidemic of typhoid fever in Atlanta. Clinical and epidemiologic observations. JAMA. 1966;197(3):179-84.
25. Colon AR, Gross DR, Tamer MA. Typhoid fever in children. Pediatrics. 1975;56(4):606-9.
26. Colon AR. Gastrointestinal phenomena in childhood typhoid fever. SouthMed J. 1976;69(7):914.
27. Daoud AS, Zaki M, Pugh RN, al-Mutairi G, Beseiso R, Nasrallah AY. Clinical presentation of enteric fever: its changing pattern in Kuwait. J TropMed Hyg. 1991;94(5):341-7
28. Del Rosario AJ, Haynes RE, Cramblett HG. Typhoid fever in children. OhioState Med J. 1971;67(6):516-20.
29. Dheer G, Kundra S, Singh T. Clinical and laboratory profile of enteric fever in children in northern India. Trop Doct. 2012 Jul;42(3):154-6.
30. Duggan MB, Beyer L. Enteric fever in young Yoruba children. ArchDisChild. 1975;50(1):67-71.

31. Dutta P, Lahiri M, Bhattacharya SK, Saha MR, Mitra U, Rasaily R, et al. Hepatitis-like presentation in typhoid fever. TransRSocTropMed Hyg. 1992;86(1):92.
32. Dutta P, Rasaily R, Saha MR, Mitra U, Manna B, Chakraborty S, et al. Randomized clinical trial of furazolidone for typhoid fever in children. ScandJGastroenterol. 1993;28(2):168-72.
33. Ellis ME, Moosa A, Hillier V. A review of typhoid fever in South African black children. PostgradMed J. 1990;66(782):1032-6..
34. Emary K, Moore CE, Chanpheaktra N, An KP, Chheng K, Sona S, et al. Enteric fever in Cambodian children is dominated by multidrug-resistant H58 Salmonella enterica serovar Typhi with intermediate susceptibility to ciprofloxacin. Trans R Soc Trop Med Hyg. 2012 Dec;106(12):718-24.
35. Galloway H, Clark NS, Blackhall M. Paediatric aspects of the Aberdeen typhoid outbreak. ArchDisChild. 1966;41(215):63-8.
36. Garg K, Mangal N, Mathur HC. Clinical profile of multi drug resistant typhoid fever in Jaipur City. Indian Pediatr. 1994;31(2):191-3.
37. Garg RA, Krashak R. Typhoid fever before two years of age. Indian Pediatr. 1993;30(6):805-8
38. Girgis NI, Tribble DR, Sultan Y, Farid Z. Short course chemotherapy with cefixime in children with multidrug-resistant Salmonella typhi Septicaemia. J TropPediatr. 1995;41(6):364-5.
39. Gulati S, Marwaha RK, Singhi S, Ayyagari A, Kumar L. Third generation cephalosporins in multi-drug resistant typhoid fever. Indian Pediatr. 1992;29(4):513-6.
40. Hardy G, Padfield CJ, Chadwick P, Partington MW. Typhoid outbreak in Kingston, Ont: experience with high-dose oral ampicillin. CanMed AssocJ. 1977;116(7):761-4, 7.
41. Hassan M. Multiply-resistant Salmonella typhi in children. Drugs. 1995;49 Suppl 2:457-9.
42. Hoa NT, Diep TS, Wain J, Parry CM, Hien TT, Smith MD, et al. Community-acquired septicaemia in southern Viet Nam: the importance of multidrug-resistant Salmonella typhi. TransRSocTropMedHyg. 1998;92(5):503-8.
43. Ispahani P, Slack RC. Enteric fever and other extraintestinal salmonellosis in University Hospital, Nottingham, UK, between 1980 and 1997. EurJ ClinMicrobiolInfectDis. 2000;19(9):679-87.
44. Jagadish K, Patwari AK, Sarin SK, Prakash C, Srivastava DK, Anand VK. Hepatic manifestations in typhoid fever. Indian Pediatr. 1994;31(7):807-11.
45. Jain S, Chitnis DS, Sham A, Rathi S, Inamdar S, Rindani GJ. Outbreak of chloramphenicol resistant typhoid fever. Indian Pediatr. 1987;24(3):193-7
46. Johnson AO, Aderele WI. Enteric fever in childhood. J TropMed Hyg. 1981;84(1):29-35.
47. Kabra SK, Madhulika, Talati A, Soni N, Patel S, Modi RR. Multidrug-resistant typhoid fever. TropDoct. 2000;30(4):195-7.
48. Kanra G, Yurdakok M, Secmeer G, Arvas A, Dereli O, Sarikayalar F, et al. Clinical findings of pediatric patients during an outbreak of typhoid fever in Ankara. TurkJ Pediatr. 1985;27(1):11-5.
49. Koul PB, Murali MV, Sharma PP, Ghai OP, Ramchandran VG, Talwar V. Multi drug resistant Salmonella typhi infection: clinical profile and therapy. Indian Pediatr. 1991;28(4):357-61.
50. Kumar R, Gupta N. Multidrug-resistant typhoid fever. Indian J Pediatr. 2007;74(1):39-42.

51. Kapoor JP, Mohan M, Talwar V, Daral TS, Bhargava SK. Typhoid fever in young children. Indian Pediatr. 1985;22(11):811-3.
52. Kulkarni ML, Rego SJ. Acute acalculous cholecystitis in typhoid fever. Indian Pediatr. 1995;32(7):829-30
53. Khan M, Coovadia YM, Connolly C, Sturm AW. Influence of sex on clinical features, laboratory findings, and complications of typhoid fever. AmJ TropMed Hyg. 1999;61(1):41-6.
54. Khuri-Bulos N. Enteric fevers in children. The importance of age in the varying clinical picture. ClinPediatr (Phila). 1981;20(7):448-52
55. Kagwa-Nyanzi JA. Typhoid fever in African children. J TropPediatr EnvironChild Health. 1971;17(3):105-9.
56. Laditan AA, Alausa KO. Problems in the clinical diagnosis of typhoid fever in children in the tropics. AnnTropPaediatr. 1981;1(3):191-5.
57. Lepage P, Bogaerts J, Van GC, Ntahorutaba M, Nsengumuremyi F, Hitimana DG, et al. Community-acquired bacteraemia in African children. Lancet. 1987;1(8548):1458-61.
58. Leung DT, Bogetz J, Itoh M, Ganapathi L, Pietroni MA, Ryan ET, et al. Factors associated with encephalopathy in patients with Salmonella enterica serotype Typhi bacteremia presenting to a diarrheal hospital in Dhaka, Bangladesh. Am J Trop Med Hyg. 2012 Apr;86(4):698-702.
59. Libov A. Enteric fever in children. Indian JPediatr. 1960;27:125-8.
60. Malik AS. Complications of bacteriologically confirmed typhoid fever in children. J TropPediatr. 2002;48(2):102-8.
61. Malik AS, Malik RH. Typhoid fever in Malaysian children. Med J Malaysia. 2001;56(4):478-90.
62. Mallouh AA, Sa'di AR. White blood cells and bone marrow in typhoid fever. Pediatr InfectDisJ. 1987;6(6):527-9.
63. Memon IA, Billoo AG, Memon HI. Cefixime: an oral option for the treatment of multidrug-resistant enteric fever in children. SouthMed J. 1997;90(12):1204-7.
64. Mishra S, Patwari AK, Anand VK, Pillai PK, Aneja S, Chandra J, et al. A clinical profile of multidrug resistant typhoid fever. Indian Pediatr. 1991;28(10):1171-4.
65. Misra S, Diaz PS, Rowley AH. Characteristics of typhoid fever in children and adolescents in a major metropolitan area in the United States. ClinInfectDis. 1997;24(5):998-
66. Moussa K. Enterica of childhood. J EgyptPublic Health Assoc. 1970;45(1):48-55.
67. Mtove G, Amos B, von Seidlein L, Hendriksen I, Mwambuli A, Kimera J, et al. Invasive salmonellosis among children admitted to a rural Tanzanian hospital and a comparison with previous studies. PLoS One. 2010;5(2):e9244.
68. Mulligan TO. Typhoid fever in young children. BrMed J. 1971;4(5788):665-7.
69. Naeemullah S. A 3 year study of children with enteric fever. Pakistan Pediatric Journal. 1994;18(4):165-9.
70. Nesbitt A, Mirza NB. Salmonella septicaemias in Kenyan children. J TropPediatr. 1989;35(1):35-9.
71. Oh HM, Masayu Z, Chew SK. Typhoid fever in hospitalized children in Singapore. J Infect. 1997;34(3):237-42.
72. Palacios Malmaceda PG, Vela Acosta JJ, Gutierez AW. [Typhoid fever in children under 2 years of age]. BolMedHospInfant Mex. 1981;38(3):473-83.

73. Papaevangelou V, Syriopoulou V, Charissiadou A, Pangalis A, Mostrou G, Theodoridou M. Salmonella bacteraemia in a tertiary children's hospital. ScandJ InfectDis. 2004;36(8):547-51.
74. Rajajee S, Anandi TB, Subha S, Vatsala BR. Patterns of resistant Salmonella typhi infection in infants. JTropPediatr. 1995;41(1):52-4.
75. Raman TS, Krishnamurthy L, Menon PK, Singh D, Jayaprakash DG. Clinical profile and therapy in enteric fever. Indian Pediatr. 1994;31(2):196-9.
76. Ramanan A, Pandit N, Yeshwanth M. Unusual complications in a multidrug resistant Salmonella typhi outbreak. Indian Pediatr. 1992;29(1):118-20.
77. Rasaily R, Dutta P, Saha MR, Mitra U, Lahiri M, Pal SC. Multi-drug resistant typhoid fever in hospitalised children. Clinical, bacteriological and epidemiological profiles. EurJ Epidemiol. 1994;10(1):41-6.
78. Rathore MH, Bux D, Hasan M. Multidrug-resistant Salmonella typhi in Pakistani children: clinical features and treatment. SouthMed J. 1996;89(2):235-7.
79. Scragg JN, Rubidge CJ. Trimethoprim and sulphamethoxazole in typhoid fever in children. BrMed J. 1971;3(5777):738-41.
80. Secmeer G, Kanra G, Cemeroglu AP, Ozen H, Ceyhan M, Ecevit Z. Salmonella typhi infections. A 10-year retrospective study. TurkJ Pediatr. 1995;37(4):339-41.
81. Sethuraman S, Mahamood M, Kareem S. Furazolidone in multi-resistant childhood typhoid fever. AnnTropPaediatr. 1994;14(4):321-4.
82. Sharma A, Gathwala G. Clinical profile and outcome in enteric fever. Indian Pediatr. 1993;30(1):47-50.
83. Shetty AK, Mital SR, Bahrainwala AH, Khubchandani RP, Kumta NB. Typhoid hepatitis in children. J TropPediatr. 1999;45(5):287-90.
84. Shwe TN, Nyein MM, Yi W, Mon A. Blood culture isolates from children admitted to Medical Unit III, Yangon Children's Hospital, 1998. Southeast Asian J TropMed Public Health.
85. Singh H, Raizada N. Chloramphenicol resistant typhoid fever. Indian Pediatr. 1991;28(4):433.
86. Smith M. Typhoid admissions in childhood. TropDoct. 1986;16(4):150-2
87. Soelistyowati S, Soenarto Y, Soesilo H, Widiarto, Widiatmodjo, Ismangoen. Typhoid fever in children. PaediatrIndones. 1982;22(7-8):138-46.
88. Stormon MO, McIntyre PB, Morris J, Fasher B. Typhoid fever in children: diagnostic and therapeutic difficulties. Pediatr InfectDisJ. 1997;16(7):713-4.
89. Su CP, Chen YC, Chang SC. Changing characteristics of typhoid fever in Taiwan. J MicrobiolImmunolInfect. 2004;37(2):109-14.
90. Takkar VP, Kumar R, Takkar R, Khurana S. Resurgence of chloramphenicol sensitive Salmonella typhi. Indian Pediatr. 1995;32(5):586-7.
91. Thambidorai CR, Shyamala J, Sarala R, Vatsala RB, Tamizhisai S. Acute acalculous cholecystitis associated with enteric fever in children. Pediatr InfectDisJ. 1995;14(9):812-3.
92. Tatli MM, Aktas G, Kosecik M, Yilmaz A. Treatment of typhoid fever in children with a flexible-duration of ceftriaxone, compared with 14-day treatment with chloramphenicol.
93. Thisyakorn U, Mansuwan P. Comparative efficacy of mecillinam, mecillinam/amoxicillin and trimethoprim-sulfamethoxazole for treatment of typhoid fever in children. PediatrInfectDisJ. 1992;11(11):979-80.
94. Thisyakorn U, Mansuwan P, Taylor DN. Typhoid and paratyphoid fever in 192 hospitalized children in Thailand. AmJ DisChild. 1987;141(8):862-5.

95. Tolaymat A, Fakhreddine F, David CB, Whitworth JM. Typhoid fever in children: a forgotten disease? SouthMed J. 1979;72(2):136-8.
96. Topley JM. Mild typhoid fever. ArchDisChild. 1986;61(2):164-7.
97. Verma PS, Mullick P, Ghosh S. Neurological manifestations of enteric fever: clinical profile and correlation with ultimate outcome. Indian Pediatr. 1972;9(11):681-5.
98. Walia M, Gaind R, Mehta R, Paul P, Aggarwal P, Kalaivani M. Current perspectives of enteric fever: a hospital-based study from India. AnnTropPaediatr. 2005;25(3):161-74.
99. Walia M, Gaind R, Paul P, Mehta R, Aggarwal P, Kalaivani M. Age-related clinical and microbiological characteristics of enteric fever in India. TransRSocTropMed Hyg. 2006;100(10):942-8.
100. Wang JL, Kao JH, Tseng SP, Teng LJ, Ho SW, Hsueh PR. Typhoid fever and typhoid hepatitis in Taiwan. EpidemiolInfect. 2005;133(6):1073-9.
101. Wongsawat J, Pancharoen C, Thisyakorn U. Typhoid fever in children: experience in King Chulalongkorn Memorial Hospital. J Med AssocThai. 2002;85(12):1247-50.
102. Yap YF, Puthucheary SD. Typhoid fever in children--a retrospective study of 54 cases from Malaysia. Singapore Med J. 1998;39(6):260-2.

- **Table S9: Typhoid fever in children in with multi-drug resistant versus sensitive strains of salmonella**

	**Multi-drug resistant** 13 studies			**Sensitive** 22 studies		
**Demographics/History**	**n**	**N**	**%**	**n**	**N**	**%**
Males	358	601	**60**	864	1500	**58**
Duration of illness 1 - 2 weeks	80	272	**29**	208	897	**23**
Duration of illness >2 weeks	83	272	**31**	164	897	**18**
Duration of illness > 1 week	208	334	**62**	407	1034	**39**
Ampicillin/Amoxycillin resistance	125	125	**100**	9	186	**5**
Co-trimoxazole resistance	125	125	**100**	14	186	**8**
**Laboratory features**						
Severe anaemia (<7.5 g/dl)	2	39	**5**	13	75	**17**
Thrombocytopenia (<150 x 10^3/µL)	3	62	**5**	39	119	**33**
**Other signs and symptoms**						
Chills or rigors	33	116	**28**	22	192	**11**
Anorexia/refusal to feed	27	77	**35**	85	253	**34**
Continuous fever	81	81	**100**	66	171	**39**
Intermittent fever	44	62	**71**	52	137	**38**
Remittent fever	11	11	**100**	51	137	**37**
Chelosis	5	112	**4**	6	161	**4**
**Other complications**						
Myocarditis	10	157	**6**	5	313	**2**
Meningitis or meningismus	3	54	**6**	6	210	**3**
Hepatitis or raised LFTs	59	413	**14**	22	210	**10**
Jaundice	43	416	**10**	12	317	**4**
Acute acalculouscholecystitis	4	80	**5**	3	122	**2**
Edema or pleural effusion or ascites	5	181	**3**	0	11	**0**
Convulsions	2	54	**4**	8	233	**3**
Epistaxis/bleeding (unspecified)	2	39	**5**	3	62	**5**
Any abscess	0	62	**0**	1	47	**2**

Renal involvement	3	95	**3**	46	116	**40**
Arthritis or arthralgia or myalgia	1	77	**1**	45	233	**19**

- Table S10: Associations of clinical features in enteric fever in children with multi-drug resistant versus sensitive strains of *S. Typhi* and *S. Paratyphi*

Clinical Features	Odds Ratio	95% Confidence Interval
Duration of illness ≤ 1week	0.4	0.3 - 0.5
Headache	2.4	1.7 - 3.3
Toxicity	2.1	1.6 - 2.6
Hepatomegaly	2.3	1.9 - 2.7
Splenomegaly	2.4	2.0- 2.9
Abdominal pain/tenderness	2.4	2.0 – 3.0
Abdominal distention or ileus	2.6	1.7 - 4.1
Anaemia	1.6	1.3 - 2.0
Leucopenia	0.5	0.3 - 0.8
Shock	2.9	1.2 - 7.3
Pneumonia	2.2	1.3 - 3.7
GI bleeding	2.3	1.1 - 4.5
Relapse	0.3	0.1 - 0.7
High grade fever	0.6	0.5 - 0.8
Bradycardia or relative bradycardia	9.1	2.3 - 36.4
Dehydration	10.9	3.3 - 36.0
Duration of illness > 1 week	2.7	2.1 - 3.4
Jaundice	2.9	1.5 - 5.7
Renal involvement	0.05	0.01 - 0.2
Duration of illness >2 weeks	2.0	1.4 - 2.7
Thrombocytopenia (<150)	0.1	0.03 - 0.3
Chills or rigors	3.1	1.7 - 5.6
Myocarditis	4.2	1.4 - 12.5
Arthritis or arthralgia or myalgia	0.05	0.01 - 0.4

References to Table 4, Table S9 and Table S10: Typhoid fever in children with multi-drug resistant versus sensitive strains of salmonella

Sensitive

1. Agarwal KS, Singh SK, Kumar N, Srivastav R, Rajkumar A. A study of current trends in enteric fever. J CommunDis. 1998;30(3):171-4.
2. Bhutta ZA. Impact of age and drug resistance on mortality in typhoid fever. ArchDisChild. 1996;75(3):214-7.
3. Bhutta ZA. Enteric Fever: Risk Factors for Treatment Failure in the Era of Multidrug Resistance. UNPUBLISHED.

4. Buch NA, Hassan MU, Kakroo DK. Enteric fever--a changing sensitivity pattern, clinical profile and outcome. Indian Pediatr. 1994;31(8):981-5.
5. Chandra R, Srinivasan S, Nalini P, Rao RS. Multidrug resistant enteric fever. J TropMed Hyg. 1992;95(4):284-7.
6. Choo KE, Razif A, Ariffin WA, Sepiah M, Gururaj A. Typhoid fever in hospitalized children in Kelantan, Malaysia. AnnTropPaediatr. 1988;8(4):207-12.
7. Hardy G, Padfield CJ, Chadwick P, Partington MW. Typhoid outbreak in Kingston, Ont: experience with high-dose oral ampicillin. CanMed AssocJ. 1977;116(7):761-4, 7.
8. Jain S, Chitnis DS, Sham A, Rathi S, Inamdar S, Rindani GJ. Outbreak of chloramphenicol resistant typhoid fever. Indian Pediatr. 1987;24(3):193-7.
9. Kabra SK, Madhulika, Talati A, Soni N, Patel S, Modi RR. Multidrug-resistant typhoid fever. TropDoct. 2000;30(4):195-7.
10. Koul PB, Murali MV, Sharma PP, Ghai OP, Ramchandran VG, Talwar V. Multi drug resistant Salmonella typhi infection: clinical profile and therapy. Indian Pediatr. 1991;28(4):357-61.
11. Lepage P, Bogaerts J, Van GC, Ntahorutaba M, Nsengumuremyi F, Hitimana DG, et al. Community-acquired bacteraemia in African children. Lancet. 1987;1(8548):1458-61.
12. Malik AS. Complications of bacteriologically confirmed typhoid fever in children. J TropPediatr. 2002;48(2):102-8.
13. Malik AS, Malik RH. Typhoid fever in Malaysian children. Med J Malaysia. 2001;56(4):478-90.
14. ishra S, Patwari AK, Anand VK, Pillai PK, Aneja S, Chandra J, et al. A clinical profile of multidrug resistant typhoid fever. Indian Pediatr. 1991;28(10):1171-4.
15. Nesbitt A, Mirza NB. Salmonella septicaemias in Kenyan children. J TropPediatr. 1989;35(1):35-9.
16. Papaevangelou V, Syriopoulou V, Charissiadou A, Pangalis A, Mostrou G, Theodoridou M. Salmonella bacteraemia in a tertiary children's hospital. ScandJ InfectDis. 2004;36(8):547-51.
17. Raman TS, Krishnamurthy L, Menon PK, Singh D, Jayaprakash DG. Clinical profile and therapy in enteric fever. Indian Pediatr. 1994;31(2):196-9.
18. Sharma A, Gathwala G. Clinical profile and outcome in enteric fever. Indian Pediatr. 1993;30(1):47-50.
19. Su CP, Chen YC, Chang SC. Changing characteristics of typhoid fever in Taiwan. J MicrobiolImmunolInfect. 2004;37(2):109-14.
20. Takkar VP, Kumar R, Takkar R, Khurana S. Resurgence of chloramphenicol sensitive Salmonella typhi. Indian Pediatr. 1995;32(5):586-7.
21. Tatli MM, Aktas G, Kosecik M, Yilmaz A. Treatment of typhoid fever in children with a flexible-duration of ceftriaxone, compared with 14-day treatment with chloramphenicol. IntJAntimicrobAgents. 2003;21(4):350-3.
22. Thisyakorn U, Mansuwan P. Comparative efficacy of mecillinam, mecillinam/amoxicillin and trimethoprim-sulfamethoxazole for treatment of typhoid fever in children. PediatrInfectDisJ. 1992;11(11):979-80.
23. Wang JL, Kao JH, Tseng SP, Teng LJ, Ho SW, Hsueh PR. Typhoid fever and typhoid hepatitis in Taiwan. EpidemiolInfect. 2005;133(6):1073-9.
24. Wongsawat J, Pancharoen C, Thisyakorn U. Typhoid fever in children: experience in King Chulalongkorn Memorial Hospital. J Med AssocThai. 2002;85(12):1247-50.

MDR

1. Bhutta ZA. Impact of age and drug resistance on mortality in typhoid fever. ArchDisChild. 1996;75(3):214-7.
2. Bhutta ZA. Enteric Fever: Risk Factors for Treatment Failure in the Era of Multidrug Resistance. UNPUBLISHED.
3. Buch NA, Hassan MU, Kakroo DK. Enteric fever--a changing sensitivity pattern, clinical profile and outcome. Indian Pediatr. 1994;31(8):981-5.
4. Chandra R, Srinivasan S, Nalini P, Rao RS. Multidrug resistant enteric fever. J TropMed Hyg. 1992;95(4):284-7.
5. Dutta P, Lahiri M, Bhattacharya SK, Saha MR, Mitra U, Rasaily R, et al. Hepatitis-like presentation in typhoid fever. TransRSocTropMed Hyg. 1992;86(1):92.
6. Girgis NI, Tribble DR, Sultan Y, Farid Z. Short course chemotherapy with cefixime in children with multidrug-resistant Salmonella typhi Septicaemia. J TropPediatr. 1995;41(6):364-5.
7. Gulati S, Marwaha RK, Singhi S, Ayyagari A, Kumar L. Third generation cephalosporins in multi-drug resistant typhoid fever. Indian Pediatr. 1992;29(4):513-6.
8. Jagadish K, Patwari AK, Sarin SK, Prakash C, Srivastava DK, Anand VK. Hepatic manifestations in typhoid fever. Indian Pediatr. 1994;31(7):807-11.
9. Kabra SK, Madhulika, Talati A, Soni N, Patel S, Modi RR. Multidrug-resistant typhoid fever. TropDoct. 2000;30(4):195-7.
10. Khan MI, Soofi SB, Ochiai RL, Khan MJ, Sahito SM, Habib MA, et al. Epidemiology, clinical presentation, and patterns of drug resistance of Salmonella Typhi in Karachi, Pakistan. J Infect Dev Ctries. 2012 Oct;6(10):704-14.
11. Koul PB, Murali MV, Sharma PP, Ghai OP, Ramchandran VG, Talwar V. Multi drug resistant Salmonella typhi infection: clinical profile and therapy. Indian Pediatr. 1991;28(4):357-61.
12. Kumar R, Gupta N. Multidrug-resistant typhoid fever. Indian J Pediatr. 2007;74(1):39-42.
13. Mishra S, Patwari AK, Anand VK, Pillai PK, Aneja S, Chandra J, et al. A clinical profile of multidrug resistant typhoid fever. Indian Pediatr. 1991;28(10):1171-4.
14. Raman TS, Krishnamurthy L, Menon PK, Singh D, Jayaprakash DG. Clinical profile and therapy in enteric fever. Indian Pediatr. 1994;31(2):196-9.
15. Rasaily R, Dutta P, Saha MR, Mitra U, Lahiri M, Pal SC. Multi-drug resistant typhoid fever in hospitalised children. Clinical, bacteriological and epidemiological profiles. EurJ Epidemiol. 1994;10(1):41-6.
16. Sharma A, Gathwala G. Clinical profile and outcome in enteric fever. Indian Pediatr. 1993;30(1):47-50.

Table S11 :Time-line of included studies on enteric fever in children

	1950-1970s 13 studies			1980-1989 21 studies			1990 – 1999 30 studies			2000-2013 12 studies		
**Demographics/History**	**N**	**N**	**%**	**n**	**N**	**%**	**n**	**N**	**%**	**n**	**N**	**%**
Blood/bone marrow Isolates	882	1113	**79**	1283	1629	**79**	4562	4941	**92**	918	918	**100**
Pre-treatment antibiotics received	23	34	**68**	137	295	**46**	1875	3283	**57**	45	227	**20**
Duration of illness≤ 1week	204	404	**50**	516	841	**61**	711	1570	**45**	82	251	**33**
Multi-drug resistant isolates	0	127	**0**	1	351	**0**	1609	3772	**43**	281	598	**47**
Fluoroquinolone resistance							12	1148	**1**	8	167	**5**
Chloramphenicol resistance	0	127	**0**	49	497	**10**	591	1042	**57**	0	72	**0**
**Signs & Symptoms: Systemic**												
Fever	662	711	**93**	1327	1399	**95**	3694	4014	**92**	731	732	**100**
High grade fever	8	70	**11**	199	385	**52**	1847	2706	**68**	247	465	**53**
Chills or rigors	22	137	**16**	39	488	**8**	185	566	**33**	48	190	**25**
Bradycardia or relative bradycardia	30	390	**8**	355	771	**46**	24	149	**16**	9	270	**3**
Anorexia/refusal to feed	203	463	**44**	269	523	**51**	706	3046	**23**	92	319	**29**
Headache	147	538	**27**	456	1235	**37**	295	3256	**9**	314	766	**41**
Toxicity	171	295	**58**	79	288	**27**	978	3310	**30**	48	76	**63**
Coated tongue	81	150	**54**	157	196	**80**	146	294	**50**	65	198	**33**
**Respiratory and abdominal**												
Cough	244	641	**38**	290	878	**33**	464	2377	**20**	178	666	**27**
Nausea or vomiting	290	655	**44**	354	935	**38**	1361	3757	**36**	282	783	**36**
Diarrhea	280	701	**40**	718	1561	**46**	1312	3964	**33**	182	802	**23**
Constipation	87	348	**25**	243	811	**30**	251	3445	**7**	35	583	**6**
Hepatomegaly	139	471	**30**	372	747	**50**	1922	4123	**47**	359	603	**60**
Splenomegaly	343	869	**39**	266	905	**29**	1280	4358	**29**	222	603	**37**
Abdominal pain or tenderness	273	559	**49**	574	1476	**39**	764	3194	**24**	383	763	**50**
Abdominal distention or ileus	235	611	**38**	292	1155	**25**	171	3657	**5**	43	515	**8**
**Laboratory features**												
Anaemia (Hb < 12 g/dl)	233	467	**50**	323	967	**33**	2124	2959	**72**	189	337	**56**
Leucopenia (< 5 x 10^3/µL)	92	379	**24**	272	906	**30**	109	1589	**7**	26	338	**8**
Leucocytosis (> 15 x 10^3/µL)	16	161	**10**	25	302	**8**	330	1465	**23**	54	275	**20**
Thrombocytopenia (<150 x 10^3/µL)	41	161	**25**	104	415	**25**	79	1188	**7**	39	198	**20**
**Complications & outcome**												
Shock or hypotension	8	188	**4**	3	81	**4**	89	2344	**4**	24	408	**6**
Altered mental status	146	650	**22**	276	1495	**18**	290	4176	**7**	75	517	**15**
Pneumonia or chest signs	117	610	**19**	276	1204	**23**	148	1580	**9**	37	394	**9**
GI bleeding	40	784	**5**	23	1351	**2**	48	716	**7**	39	586	**7**
GI perforation	58	660	**9**	12	1047	**1**	7	775	**1**	3	130	**2**
Hepatitis or raised LFTs	18	277	**6**	66	145	**46**	172	2072	**8**	43	266	**16**

Jaundice	21	484	**4**	22	534	**4**	81	3381	**2**	38	408	**9**
Relapse	59	658	**9**	29	1053	**3**	173	3235	**5**	5	248	**2**
Death	73	986	**7**	40	1518	**3**	62	4288	**1**	5	722	**1**

References to Table S6 : Time-line of included studies on enteric fever in children

1950 - 1970

1. Collins RN, Marine WM, Nahmias AJ. The 1964 epidemic of typhoid fever in Atlanta. Clinical and epidemiologic observations. JAMA. 1966;197(3):179-84.
2. Colon AR. Gastrointestinal phenomena in childhood typhoid fever. SouthMed J. 1976;69(7):914.
3. Colon AR, Gross DR, Tamer MA. Typhoid fever in children. Pediatrics. 1975;56(4):606-9.
4. Del Rosario AJ, Haynes RE, Cramblett HG. Typhoid fever in children. OhioState Med J. 1971;67(6):516-20.
5. Duggan MB, Beyer L. Enteric fever in young Yoruba children. ArchDisChild. 1975;50(1):67-71.
6. Galloway H, Clark NS, Blackhall M. Paediatric aspects of the Aberdeen typhoid outbreak. ArchDisChild. 1966;41(215):63-8.
7. Hardy G, Padfield CJ, Chadwick P, Partington MW. Typhoid outbreak in Kingston, Ont: experience with high-dose oral ampicillin. CanMed AssocJ. 1977;116(7):761-4, 7.
8. Johnson AO, Aderele WI. Enteric fever in childhood. J TropMed Hyg. 1981;84(1):29-35.
9. Laditan AA, Alausa KO. Problems in the clinical diagnosis of typhoid fever in children in the tropics. AnnTropPaediatr. 1981;1(3):191-5.
10. Khuri-Bulos N. Enteric fevers in children. The importance of age in the varying clinical picture. ClinPediatr (Phila). 1981;20(7):448-52.
11. Kagwa-Nyanzi JA. Typhoid fever in African children. J TropPediatr EnvironChild Health. 1971;17(3):105-9.
12. Moussa K. Enterica of childhood. J EgyptPublic Health Assoc. 1970;45(1):48-55.
13. Mulligan TO. Typhoid fever in young children. BrMed J. 1971;4(5788):665-7.
14. Scragg JN, Rubidge CJ. Trimethoprim and sulphamethoxazole in typhoid fever in children. BrMed J. 1971;3(5777):738-41.
15. Tolaymat A, Fakhreddine F, David CB, Whitworth JM. Typhoid fever in children: a forgotten disease? SouthMed J. 1979;72(2):136-8.
16. Verma PS, Mullick P, Ghosh S. Neurological manifestations of enteric fever: clinical profile and correlation with ultimate outcome. Indian Pediatr. 1972;9(11):681-5.

1980 - 1989

1. Brian MJ. Typhoid in children in Goroka. PNGMed J. 1990;33(1):68-9.
2. Caglar MK, Ozsoylu S, Kanra G. Relative granulocytosis in childhood typhoid fever. J Pediatr. 1983;102(4):603-4.
3. Chiu CH, Lin TY. Typhoid fever in children. Lancet. 1999;354(9194):2001-2.

4. Chiu CH, Tsai JR, Ou JT, Lin TY. Typhoid fever in children: a fourteen-year experience. Acta PaediatrTaiwan. 2000;41(1):28-32.
5. Choo KE, Razif A, Ariffin WA, Sepiah M, Gururaj A. Typhoid fever in hospitalized children in Kelantan, Malaysia. AnnTropPaediatr. 1988;8(4):207-12.
6. Chow CB, Wang PS, Leung NK. Typhoid fever in Hong Kong children. AustPaediatrJ. 1989;25(3):147-50.
7. Daoud AS, Zaki M, Pugh RN, al-Mutairi G, Beseiso R, Nasrallah AY. Clinical presentation of enteric fever: its changing pattern in Kuwait. J TropMed Hyg. 1991;94(5):341-7.
8. Ellis ME, Moosa A, Hillier V. A review of typhoid fever in South African black children. PostgradMed J. 1990;66(782):1032-6.
9. Ferreccio C, Levine MM, Manterola A, Rodriguez G, Rivara I, Prenzel I, et al. Benign bacteremia caused by Salmonella typhi and paratyphi in children younger than 2 years. J Pediatr. 1984;104(6):899-901.
10. Green SD, Cheesbrough JS. Salmonella bacteraemia among young children at a rural hospital in western Zaire. AnnTropPaediatr. 1993;13(1):45-53.
11. Ispahani P, Slack RC. Enteric fever and other extraintestinal salmonellosis in University Hospital, Nottingham, UK, between 1980 and 1997. EurJ ClinMicrobiolInfectDis. 2000;19(9):679-87.
12. Jain S, Chitnis DS, Sham A, Rathi S, Inamdar S, Rindani GJ. Outbreak of chloramphenicol resistant typhoid fever. Indian Pediatr. 1987;24(3):193-7.
13. Kanra G, Yurdakok M, Secmeer G, Arvas A, Dereli O, Sarikayalar F, et al. Clinical findings of pediatric patients during an outbreak of typhoid fever in Ankara. TurkJ Pediatr. 1985;27(1):11-5.
14. Kapoor JP, Mohan M, Talwar V, Daral TS, Bhargava SK. Typhoid fever in young children. Indian Pediatr. 1985;22(11):811-3.
15. Lepage P, Bogaerts J, Van GC, Ntahorutaba M, Nsengumuremyi F, Hitimana DG, et al. Community-acquired bacteraemia in African children. Lancet. 1987;1(8548):1458-61.
16. Mallouh AA, Sa'di AR. White blood cells and bone marrow in typhoid fever. Pediatr InfectDisJ. 1987;6(6):527-9
17. Nesbitt A, Mirza NB. Salmonella septicaemias in Kenyan children. J TropPediatr. 1989;35(1):35-9.
18. Secmeer G, Kanra G, Cemeroglu AP, Ozen H, Ceyhan M, Ecevit Z. Salmonella typhi infections. A 10-year retrospective study. TurkJ Pediatr. 1995;37(4):339-41.
19. Soelistyowati S, Soenarto Y, Soesilo H, Widiarto, Widiatmodjo, Ismangoen. Typhoid fever in children. PaediatrIndones. 1982;22(7-8):138-46.
20. Stormon MO, McIntyre PB, Morris J, Fasher B. Typhoid fever in children: diagnostic and therapeutic difficulties. Pediatr InfectDisJ. 1997;16(7):713-4.
21. Thisyakorn U, Mansuwan P, Taylor DN. Typhoid and paratyphoid fever in 192 hospitalized children in Thailand. AmJ DisChild. 1987;141(8):862-5.
22. Yap YF, Puthucheary SD. Typhoid fever in children--a retrospective study of 54 cases from Malaysia. Singapore Med J. 1998;39(6):260-2.

1990 - 1994

1. Ahmad KA, Khan LH, Roshan B, Bhutta ZA. Factors associated with typhoid relapse in the era of multiple drug resistant strains. J Infect Dev Ctries. 2011 Oct;5(10):727-31.

2. Arora RK, Gupta A, Joshi NM, Kataria VK, Lall P, Anand AC. Multidrug resistant typhoid fever: study of an outbreak in Calcutta. Indian Pediatr. 1992;29(1):61-6.
3. Bhutta ZA. Impact of age and drug resistance on mortality in typhoid fever. ArchDisChild. 1996;75(3):214-7.
4. Bhutta ZA. Fulminant hepatic failure with typhoid fever in childhood. J Pak Med Assoc. 1991;41(6):123-6.
5. Bhutta ZA, Mansoorali N, Hussain R. Plasma cytokines in paediatric typhoidal salmonellosis: correlation with clinical course and outcome. J Infect. 1997;35(3):253-6.
6. Bhutta ZA, Naqvi SH. Hepatic dysfunction in paediatric typhoidal salmonellosis. AnnTropPaediatr. 1991;11(3):203-5.
7. Bhutta ZA, Naqvi SH, Durrani S, Suria A. Chloramphenicol therapy of typhoid fever and its relationship to hepatic dysfunction. JTropPediatr. 1991;37(6):320-2.
8. Bhutta ZA, Naqvi SH, Razzaq RA, Farooqui BJ. Multidrug-resistant typhoid in children: presentation and clinical features. RevInfectDis. 1991;13(5):832-6.
9. Bhutta ZA, Niazi SK, Suria A. Chloramphenicol clearance in typhoid fever: implications for therapy. Indian J Pediatr. 1992;59(2):213-9
10. Bhutta ZA. Enteric Fever: Risk Factors for Treatment Failure in the Era of Multidrug Resistance. UNPUBLISHED.
11. Biswal N. Neurological manifestations of typhoid fever in children. J TropPediatr. 1994;40(3):190.
12. Biswal N, Mathai B, Bhatia BD, Srinivasan S, Nalini P. Enteric fever: a changing perspective. Indian Pediatr. 1994;31(7):813-9.
13. Buch NA, Hassan MU, Kakroo DK. Enteric fever--a changing sensitivity pattern, clinical profile and outcome. Indian Pediatr. 1994;31(8):981-5.
14. Chandra R, Srinivasan S, Nalini P, Rao RS. Multidrug resistant enteric fever. J TropMed Hyg. 1992;95(4):284-7.
15. Dutta P, Lahiri M, Bhattacharya SK, Saha MR, Mitra U, Rasaily R, et al. Hepatitis-like presentation in typhoid fever. TransRSocTropMed Hyg. 1992;86(1):92.
16. Dutta P, Rasaily R, Saha MR, Mitra U, Manna B, Chakraborty S, et al. Randomized clinical trial of furazolidone for typhoid fever in children. ScandJGastroenterol. 1993;28(2):
17. Garg RA, Krashak R. Typhoid fever before two years of age. Indian Pediatr. 1993;30(6):805-8.
18. Girgis NI, Tribble DR, Sultan Y, Farid Z. Short course chemotherapy with cefixime in children with multidrug-resistant Salmonella typhi Septicaemia. J TropPediatr. 1995;41(6):364-5.
19. Hassan M. Multiply-resistant Salmonella typhi in children. Drugs. 1995;49 Suppl 2:457-9.
20. Hoa NT, Diep TS, Wain J, Parry CM, Hien TT, Smith MD, et al. Community-acquired septicaemia in southern Viet Nam: the importance of multidrug-resistant Salmonella typhi. TransRSocTropMedHyg. 1998;92(5):503-8.
21. Khan M, Coovadia YM, Connolly C, Sturm AW. Influence of sex on clinical features, laboratory findings, and complications of typhoid fever. AmJ TropMed Hyg. 1999;61(1):41-6.
22. Koul PB, Murali MV, Sharma PP, Ghai OP, Ramchandran VG, Talwar V. Multi drug resistant Salmonella typhi infection: clinical profile and therapy. Indian Pediatr. 1991;28(4):357-61.

23. Memon IA, Billoo AG, Memon HI. Cefixime: an oral option for the treatment of multidrug-resistant enteric fever in children. SouthMed J. 1997;90(12):1204-7.
24. Misra S, Diaz PS, Rowley AH. Characteristics of typhoid fever in children and adolescents in a major metropolitan area in the United States. ClinInfectDis. 1997;24(5):998-1000.
25. Mishra S, Patwari AK, Anand VK, Pillai PK, Aneja S, Chandra J, et al. A clinical profile of multidrug resistant typhoid fever. Indian Pediatr. 1991;28(10):1171-4.
26. Oh HM, Masayu Z, Chew SK. Typhoid fever in hospitalized children in Singapore. J Infect. 1997;34(3):237-42.
27. Rajajee S, Anandi TB, Subha S, Vatsala BR. Patterns of resistant Salmonella typhi infection in infants. JTropPediatr. 1995;41(1):52-4.
28. Ramanan A, Pandit N, Yeshwanth M. Unusual complications in a multidrug resistant Salmonella typhi outbreak. Indian Pediatr. 1992;29(1):118-20.
29. Rasaily R, Dutta P, Saha MR, Mitra U, Lahiri M, Pal SC. Multi-drug resistant typhoid fever in hospitalised children. Clinical, bacteriological and epidemiological profiles. EurJ Epidemiol. 1994;10(1):41-6
30. Rathore MH, Bux D, Hasan M. Multidrug-resistant Salmonella typhi in Pakistani children: clinical features and treatment. SouthMed J. 1996;89(2):235-7.
31. Shetty AK, Mital SR, Bahrainwala AH, Khubchandani RP, Kumta NB. Typhoid hepatitis in children. J TropPediatr. 1999;45(5):287-90
32. Sethuraman S, Mahamood M, Kareem S. Furazolidone in multi-resistant childhood typhoid fever. AnnTropPaediatr. 1994;14(4):321-4.
33. Thambidorai CR, Shyamala J, Sarala R, Vatsala RB, Tamizhisai S. Acute acalculous cholecystitis associated with enteric fever in children. Pediatr InfectDisJ. 1995;14(9):812-3.

1995 - 1999

1. Ciftci E, Guriz H, Derya AA, Ince E, Erdem B, Dogru U. Salmonella bacteraemia in Turkish children: 37 cases seen in a university hospital between 1993 and 2002. AnnTropPaediatr. 2004;24(1):75-80.
2. Malik AS. Complications of bacteriologically confirmed typhoid fever in children. J TropPediatr. 2002;48(2):102-8.
3. Malik AS, Malik RH. Typhoid fever in Malaysian children. Med J Malaysia. 2001;56(4):478-90.
4. Papaevangelou V, Syriopoulou V, Charissiadou A, Pangalis A, Mostrou G, Theodoridou M. Salmonella bacteraemia in a tertiary children's hospital. ScandJ InfectDis. 2004;36(8):547-51.
5. Shwe TN, Nyein MM, Yi W, Mon A. Blood culture isolates from children admitted to Medical Unit III, Yangon Children's Hospital, 1998. Southeast Asian J TropMed Public Health. 2002;33(4):764-71.
6. Sinha A, Sazawal S, Kumar R, Sood S, Reddaiah VP, Singh B, et al. Typhoid fever in children aged less than 5 years. Lancet. 1999;354(9180):734-7.
7. Su CP, Chen YC, Chang SC. Changing characteristics of typhoid fever in Taiwan. J MicrobiolImmunolInfect. 2004;37(2):109-14.
8. Wongsawat J, Pancharoen C, Thisyakorn U. Typhoid fever in children: experience in King Chulalongkorn Memorial Hospital. J Med AssocThai. 2002;85(12):1247-50.

2000 onwards

1. Dheer G, Kundra S, Singh T. Clinical and laboratory profile of enteric fever in children in northern India. Trop Doct. 2012 Jul;42(3):154-6.
2. Emary K, Moore CE, Chanpheaktra N, An KP, Chheng K, Sona S, et al. Enteric fever in Cambodian children is dominated by multidrug-resistant H58 Salmonella enterica serovar Typhi with intermediate susceptibility to ciprofloxacin. Trans R Soc Trop Med Hyg. 2012 Dec;106(12):718-24.
3. Khan MI, Soofi SB, Ochiai RL, Khan MJ, Sahito SM, Habib MA, et al. Epidemiology, clinical presentation, and patterns of drug resistance of Salmonella Typhi in Karachi, Pakistan. J Infect Dev Ctries. 2012 Oct;6(10):704-14.
4. Kumar R, Gupta N. Multidrug-resistant typhoid fever. Indian J Pediatr. 2007;74(1):39-42.
5. Leung DT, Bogetz J, Itoh M, Ganapathi L, Pietroni MA, Ryan ET, et al. Factors associated with encephalopathy in patients with Salmonella enterica serotype Typhi bacteremia presenting to a diarrheal hospital in Dhaka, Bangladesh. Am J Trop Med Hyg. 2012 Apr;86(4):698-702.
6. Mtove G, Amos B, von Seidlein L, Hendriksen I, Mwambuli A, Kimera J, et al. Invasive salmonellosis among children admitted to a rural Tanzanian hospital and a comparison with previous studies. PLoS One. 2010;5(2):e9244.
7. Owais A, Sultana S, Zaman U, Rizvi A, Zaidi AK. Incidence of typhoid bacteremia in infants and young children in southern coastal Pakistan. Pediatr Infect Dis J. 2010 Nov;29(11):1035-9.
8. Pradhan R, Shrestha U, Gautam SC, Thorson S, Shrestha K, Yadav BK, et al. Bloodstream infection among children presenting to a general hospital outpatient clinic in urban Nepal. PLoS One. 2012;7(10):e47531
9. Siddiqui FJ, Rabbani F, Hasan R, Nizami SQ, Bhutta ZA. Typhoid fever in children: some epidemiological considerations from Karachi, Pakistan. Int J InfectDis. 2006;10(3):215-22.
10. Tatli MM, Aktas G, Kosecik M, Yilmaz A. Treatment of typhoid fever in children with a flexible-duration of ceftriaxone, compared with 14-day treatment with chloramphenicol. IntJAntimicrobAgents. 2003;21(4):350-3.
11. Walia M, Gaind R, Mehta R, Paul P, Aggarwal P, Kalaivani M. Current perspectives of enteric fever: a hospital-based study from India. AnnTropPaediatr. 2005;25(3):161-74.
12. Walia M, Gaind R, Paul P, Mehta R, Aggarwal P, Kalaivani M. Age-related clinical and microbiological characteristics of enteric fever in India. TransRSocTropMed Hyg. 2006;100(10):942-8.
13. Wang JL, Kao JH, Tseng SP, Teng LJ, Ho SW, Hsueh PR. Typhoid fever and typhoid hepatitis in Taiwan. EpidemiolInfect. 2005;133(6):1073-9.

Chart 1 : Definitions of variables used

Variables	As defined by author, or :
Multi-drug resistance	Resistance to all 3 of chloramphenicol, ampicillin/amoxycillin, cotrimoxazole
Weakness or malaise	fatigue, weakness, dizziness, malaise, prostration
High grade fever	> 104 F or > 40 C, hyperpyrexia, extreme hyperpyrexia
Toxicity	toxemia or ‘enteric state’
Chelosis	stomatitis, mouth ulcers, apthous ulcers, parotitis, stomatitis, cracked lips
Pneumonia, bronchopneumonia or chest signs	abnormal CXR, congestion, consolidation, rales, rhonchi, crepts, dyspnea, respiratory/chest/lung/bronchitic signs, pulmonary infiltration, bronchitis, respiratory distress, bronchial breathing, broncho-pulmonary involvement
**Complications**	
Altered mental status	mental change, encephalopathy, encephalitis, drowsy, lethargy, confusion, coma, semi-coma, delirium, psychosis, irritability, disoriented, stupor, obtundation, disordered thought, CNS disturbances, neuro-psychiatric features/manifestations, altered sensorium, clouded/altered consciousness, acute schizophrenia, global cerebral dysfunction, somnolence, apathy
Focal neurological signs or symptoms	aphasia, ataxia, deafness, motor paresis, hemiplegia, tremor, difficulty speaking, nystagmus, dysarthria, slurred speech, chorea/choreoform movements, cerebellar ataxia, cerebellitis, cerebellar disease, Parkinsonism, facial palsy, unsteady gait, unsteady/falls, myelitis, transverse myelitis, CN palsy
Hepatitis or raised LFTs	elevated SGPT/ALT, hepatic dysfunction, raised LFTs or transaminases (if not disaggregated, both ALT and/or AST elevated extracted), hepato-biliary involvement (taken ALT raised as > 35 if not specified by author), or ALT and/or bilirubin elevation, liver failure
Abdominal distention or ileus	tympanities, tympanic abdomen, meteorism, tumid abdomen, decreased or absent bowel sounds, signs of intestinal obstruction
Renal involvement	nephritis, nephrotic, proteinuria, albuminuria, raised BUN or Cr, abnormal urine d/r, hematuria, pyuria, renal failure, oliguria, nephropathy
Arthritis, arthralgia, myalgia	limb/extremity pain, leg pain, backache, bone pain, joint pain, joint swelling, reactive arthritis, bodyache, muscle pain
**Outcome**	
Relapse	Clinical, or clinical and culture-confirmed
**Laboratory features**	
Anaemia(<12g/dl)	undefined, moderate anaemia, Hb 5-10, 8.5-10Hct< 30%/32%, Hb<9, < 10, <=10.5, <= 11, < 12, <=12
Severe anaemia (<7.5 g/dl)	undefined, <5, < 6, <6.5, < 6.7, < 7.4
Leucopenia (<5 x 10^3/µL)	<4, <4.5, <3, or undefined
Leucocytosis (>15 x 10^3/µL)	or undefined
Thrombocytopenia (<150 x 10^3/µL)	<100, or undefined
Severe thrombocytopenia (<50 x 10^3/µL)	or undefined
Neutrophilia (>7.5 x 10^3/µL)	or undefined
